# From Single Markers to Polygenic Panels: A Systematic Review of Genetics in Athletic Performance

**DOI:** 10.3390/sports14100461

**Published:** 2026-10-16

**Authors:** Samuel Fernández-Lorenzo, Cristian Marín-Pagán, Pedro Emilio Alcaraz, Javier Escobar Cubiella, Francisco Javier Martínez Noguera

**Affiliations:** 1 Sabartech S.L., Science Park of the University of Valencia, 46980 Paterna, Spain; javier.escobar@sabartech.com; 2 High Performance Sports Research Centre, Universidad Católica San Antonio de Murcia, 30107 Murcia, Spain; cmarin@ucam.edu (C.M.-P.); palcaraz@ucam.edu (P.E.A.)

**Keywords:** athletic performance, genetic polymorphism, genome-wide association study, sports genomics, systematic review

## Abstract

Genetic factors contribute to variability in athletic performance among individuals, although their overall impact and practical relevance remain unclear. This systematic review aimed to provide an updated synthesis of genetic variation associated with athlete status and performance-related phenotypes. PubMed and Web of Science were searched for studies published between 1998 and 1 March 2026 examining athlete status, physical performance, training response, cardiorespiratory fitness, sport-related injury, and related phenotypes using candidate-gene, genome-wide, sequencing-based, or polygenic approaches. Study selection, data extraction, and reporting followed PRISMA 2020, and reporting completeness was assessed using a prespecified STROBE/STREGA-based checklist. In total, 204 studies were included. *ACE* insertion/deletion and *ACTN3* rs1815739 remained the most extensively investigated candidate variants, but their associations were heterogeneous across phenotypes and populations. Only two athlete-status loci, *MYBPC3* rs1052373 and *GALNT13* rs10196189, were supported by replicated genome-wide evidence, and 71% of studies included European-ancestry cohorts. Total-genotype-score (TGS) and polygenic-score (PGS) approaches better reflected the polygenic architecture of performance, although external validation and transferability across populations remained limited. Overall, individual variants showed modest, context-dependent effects, and current genetic evidence is insufficient for individual talent identification, injury prediction, or training prescription.

## 1. Introduction

Although many athletes train to reach the elite level of sport, only a few achieve this recognition, which raises the question of what factors are decisive in this sporting ascent. It is known that an athlete’s performance is influenced by a multitude of factors, such as training, nutrition, rest, lifestyle and also genetics, whose impact in terms of heritability can be as high as 68% [1].

Over the last two decades, a large number of polymorphisms have been identified that have an impact on athletic performance and which, to a greater or lesser extent, could genetically determine the status of elite athletes [2]. Some of the most studied markers are the I/D-*ACE* (angiotensin-converting enzyme gene) and R577X-*ACTN3* (actinin alpha 3) polymorphisms, which have been linked to resistance and power phenotypes, respectively [3,4]. In addition, specific genetic profiles have been associated with athletic performance and other related aspects, where specific variants in genes such as *CKM* (encoding the muscle isoform of creatine kinase), *HIF1A* (encoding the alpha subunit of hypoxia-inducible factor 1), *ACE* or *AQP1* (aquaporin 1 gene) are more prevalent in individuals with better performance [5].

It should be noted that most sports can be placed on a continuum ranging from disciplines in which strength/power predominates (weightlifting, throwing, sprinting, etc.) to disciplines in which endurance predominates (long-distance running, cycling, rowing, etc.), with many team sports and others presenting mixed or intermittent demands. The differences in physiological demands and requirements provide a justification for exploring whether certain genetic variants are differentially associated with success in specific sports or with specific performance-related phenotypes, such as strength, power, endurance, or injury risk [6]. Based on this classification, several authors have described various panels of markers with a possible impact on a sport included in one of these categories [7,8]. Technological advances, including genome-wide association studies (GWAS), high-throughput genotyping, and advances in bioinformatics, have made it possible to move beyond individual candidate genes and explore polygenic profiles associated with performance [9]. Polygenic scores that aggregate the effects of multiple variants have been proposed as potential tools for predicting training capacity or elite status [10]. However, current studies often include relatively small and ethnically homogeneous samples, use diverse and sometimes poorly standardized performance outcomes, and show limited replication across cohorts. As a result, the practical utility of genetic profiling in sport remains uncertain [11].

On the other hand, due to the characteristics of the study population, in this case high-performance athletes, the lack of samples often presents an obstacle to obtaining information that would allow progress in the characterization of genotypes and the discovery of new variants of interest. This also complicates the replication of results on the association of variants with performance, which, added to the complexity of defining and obtaining specific and valid phenotypes, hinders and delays the obtaining of solid evidence and the interpretation of results [12]. Nonetheless, advances in whole-genome sequencing, artificial intelligence, and integrative omics approaches (including transcriptomics, epigenetics, and metabolomics) are expected to enrich our understanding of sports genomics in the coming years, moving the field beyond single-marker studies and towards systems biology frameworks [13].

Taking all of the above into account, the objective of this article will be to review the literature in order to obtain a comprehensive panel of markers in which those polymorphisms with the strongest evidence in the field of athletic performance can be identified. Compared with previous broad narrative updates of the field [7], the present review was prospectively registered and conducted according to PRISMA 2020, and it includes studies published up to March 2026, considers training-response, cardiorespiratory-fitness and sport-related injury phenotypes, and grades the strength of the available evidence according to study design and replication.

## 2. Materials and Methods

### 2.1. Search Strategy

For this systematic review, we investigated human genetic variation in relation to athlete status and sport- and exercise-related phenotypes, including physical performance, training response, cardiorespiratory fitness, and susceptibility to sport-related injury. The searches included articles published between 1998 (publication of the first study on polymorphism and its impact on performance) and 1 March 2026. PubMed and Web of Science were searched using combinations of terms related to sport and athletic performance and terms related to genetic variation (e.g., “sport”, “athlete”, “strength”, “endurance”, “gene”, “variant”, “polymorphism”) in titles and abstracts (PubMed) or titles (Web of Science). Six PubMed search strategies and one Web of Science strategy were used; three of the PubMed strategies, targeting genome-wide, polygenic, cardiorespiratory-fitness and elite-athlete association studies, were added during the revision process. The last search was performed on 5 March 2026.

Additional records were identified through manual reference-list and citation searching. The database searches and record-identification process were independently replicated by two researchers. The complete search strings, Boolean operators, filters, search dates, and outputs for each individual search are provided in Supplementary Material S2.

The review was conducted and reported in accordance with the PRISMA 2020 statement [14].

### 2.2. Study Selection

**Figure 1:**
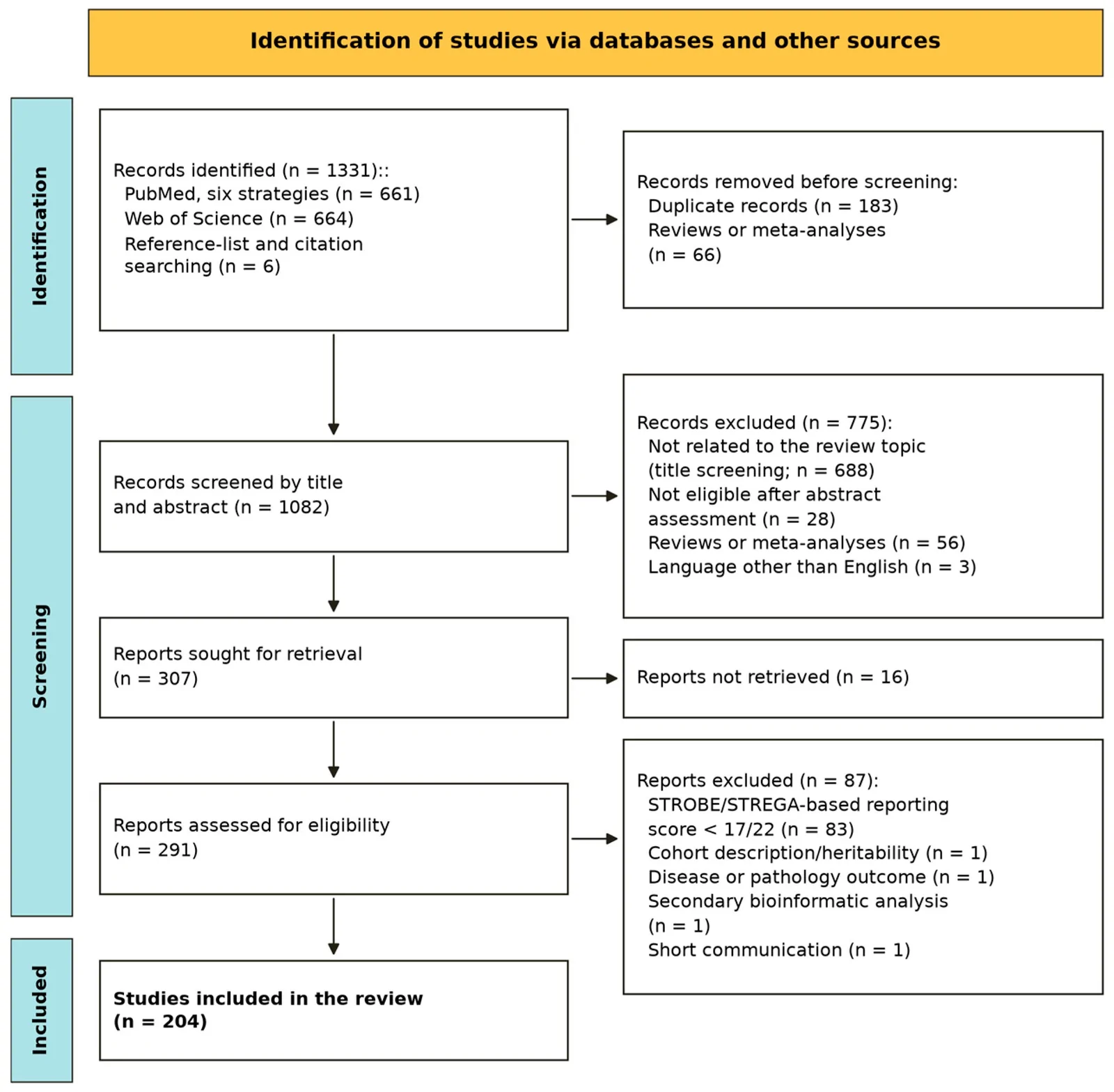
PRISMA 2020 flow diagram of study selection.

Records retrieved from the different searches were combined and duplicates were removed. Reviews or meta-analyses identified subsequently during title and abstract screening were excluded at that stage. The remaining records were screened by title and abstract against the predefined eligibility criteria. Full-text reports were subsequently sought for potentially eligible studies or whenever eligibility could not be determined reliably from the title and abstract alone. Studies for which the full text could not be retrieved were recorded separately as reports not retrieved. Title and abstract screening and subsequent full-text eligibility assessment were performed independently by two reviewers. Disagreements were resolved by consensus. Full-text reports were then assessed against the eligibility criteria, and reasons for exclusion were documented (for more details, see Figure 1). Detailed screening records are provided in Supplementary Material S2.

### 2.3. Inclusion and Exclusion Criteria

Eligible studies included the following broad methodological categories:

1. Candidate-gene association studies, including comparisons between athletes and non-athletes or between athletes from different performance or sporting groups.
2. Genotype–phenotype and training-response studies investigating associations between genetic variants and quantitative performance-related traits, including strength, power, endurance, cardiorespiratory fitness, VO_2_max/VO_2_peak, muscle-related phenotypes, or responses to exercise training.
3. Sport-related injury studies investigating genetic susceptibility to musculoskeletal injury, muscle damage, tendinopathy, ligament injury, or other injury-related phenotypes relevant to sports participation.
4. Genome-wide, exome-wide, whole-genome, whole-exome, or other sequencing-based association studies investigating athlete status or eligible performance-related phenotypes.
5. Polygenic and multi-variant studies, including polygenic scores, total genotype scores (TGS), weighted genotype scores, and related genetic-profile approaches examining athlete status, physical performance, training response, or cardiorespiratory fitness.

No specific restrictions were imposed on sex, age, or ancestry. Studies examining supplementation were not excluded per se when the primary analysis assessed whether genetic variation modified a sport- or exercise-performance response. Similarly, injury-related studies were eligible when they specifically investigated genetic susceptibility to a sport-related injury phenotype.

We only included human studies published in English with sample sizes ≥20. Meta-analyses, systematic reviews, and bibliographic reviews were not considered. Conference abstracts, letters, comments, or interviews that may have been present in the reviewed databases were also not taken into account.

We excluded studies lacking methodological detail, complete genotypic data or relevant performance outcomes; in case of doubt after title/abstract screening, the full text was reviewed.

### 2.4. Data Extraction

The following information was recorded, where available: (i) first author and year of publication; (ii) PubMed identifier (PMID) or, when unavailable, digital object identifier (DOI); (iii) study design and genetic approach; (iv) genes and genetic variants or markers investigated; (v) study population and ancestry as reported by the original authors; (vi) sex of participants; (vii) sample size; (viii) sport or exercise discipline; (ix) performance-, training-, fitness-, or injury-related phenotype investigated; (x) genotypic or allelic frequencies, where reported; and (xi) the main genetic findings.

Genome-wide or study-defined significant loci were recorded together with relevant replication information when available. For polygenic or total-genotype-score studies, the type of genetic score, the variants included in the score when reported, and the principal association with the investigated phenotype were recorded.

The complete gene-by-gene extraction matrix, including study identification, PMID/DOI, population characteristics, sport discipline, genotype/allele frequencies and main findings, is provided in Supplementary Material S1. A complete list of all included studies and the polymorphisms analyzed in each study is provided in Supplementary Material S3.

Data extraction was performed independently by two reviewers using a standardized extraction form.

### 2.5. Reporting Completeness Assessment

Reporting completeness was assessed using a 22-item checklist derived from the STROBE statement and its STREGA extension for genetic association studies [15]. Studies scoring fewer than 17 of 22 items were excluded from the final synthesis. The assessment was performed independently by two reviewers using a standardized form. Disagreements were resolved by consensus.

### 2.6. Evidence Synthesis and Interpretation

Gene–variant–phenotype associations were classified using a simple three-tier, design-based evidence framework. Tier 1 (exploratory evidence) comprised associations derived from single-cohort candidate-gene, genotype–phenotype, training-response, or non-externally validated multi-variant studies without independent replication. Tier 2 (strengthened evidence) comprised findings supported by independent replication, consistent multicohort evidence, or convergent candidate-gene evidence across independent studies and meta-analyses; genome- or exome-wide significant findings without independent external replication were also considered within this tier. Tier 3 (higher-strength evidence) comprised genome- or exome-wide significant associations replicated in an independent cohort, as well as polygenic models validated in independent external samples. Sample size, correction for multiple testing, consistency across ancestry groups, and phenotype comparability were additionally considered when interpreting findings within each tier. Evidence tier reflects methodological strength and degree of replication, not uniformity of effect direction; consistency of the associated allele, phenotype domain, and direction of effect were evaluated separately. The same hierarchy was considered when interpreting null findings.

### 2.7. Ethical Approval

Approval of this study by an ethics committee was not necessary because it is a systematic review of previously published research and does not involve the collection of new data from human participants.

### 2.8. Protocol Registration and Reporting

The review protocol was prospectively registered in the International Platform of Registered Systematic Review and Meta-analysis Protocols (INPLASY; registration no. INPLASY202630012).

The supplementary searches performed during the revision process constituted an expansion of the original search strategy designed to improve its sensitivity, particularly for genome-wide, sequencing-based, cardiorespiratory-fitness, and polygenic studies. These supplementary searches covered the same publication period and the same core review question and target phenotype domains as the original strategy. In addition, the design-based evidence-tier framework (Section 2.6) and the grouping of population descriptors into ancestry categories (Section 3.2) were introduced during the revision process and were not part of the registered protocol.

Supplementary Material S2 contains the complete database search strategies, Boolean operators, search outputs, and screening records. Supplementary Material S3 provides the characteristics of the studies included in the review and the list of identified markers, and Supplementary Material S1 summarizes the genetic variants and associated findings.

## 3. Results

### 3.1. Search Results

The search strategy identified 1331 records: 661 from PubMed, 664 from Web of Science, and 6 through manual reference-list or citation searching. After removal of 183 duplicate records and 66 reviews or meta-analyses identified before primary-study screening, 1082 records remained for title and abstract screening.

A total of 775 records were excluded during title and abstract screening, including 688 records excluded on the basis of title, 28 following abstract assessment, 56 additional reviews or meta-analyses, and 3 articles published in languages other than English. Consequently, 307 reports were sought for full-text retrieval. Sixteen reports could not be retrieved, leaving 291 full-text articles for eligibility assessment.

Of these, 87 were excluded. Eighty-three did not meet the prespecified STROBE/STREGA-based reporting-completeness threshold, and four were excluded after full-text assessment because they did not meet the remaining eligibility criteria. A total of 204 studies were therefore included in the final review (Figure 1).

The complete search strategies, database outputs, screening process, and reasons for exclusion are provided in Supplementary Material S2.

### 3.2. Characteristics of the Included Studies

The 204 included studies were published between 1999 and 2026 and encompassed a wide range of study populations, sporting disciplines, performance phenotypes, and genetic approaches. Sample sizes varied substantially, ranging from small experimental or candidate-gene studies involving approximately 20 participants to population-scale genetic studies comprising several hundred thousand individuals.

Most studies included both male and female participants (123 studies), whereas 70 included male participants only and 7 included female participants only. Three studies comprised multiple cohorts with differing sex compositions, and sex was not specified in one study.

The populations investigated were also heterogeneous. To summarise their ancestral composition, the population descriptors reported by the original authors were grouped following the ancestry categories of the GWAS Catalog [16]. Overall, 145 studies (71.1%) included at least one cohort of European or self-described Caucasian ancestry, and 129 (63.2%) were conducted exclusively in such cohorts. East Asian cohorts were included in 38 studies (18.6%), Hispanic/Latin American (mainly Brazilian) cohorts in 12 (5.9%), Greater Middle Eastern (Turkish) cohorts in 8 (3.9%), African or African-diaspora cohorts in 4 (2.0%), and Central Asian (Kazakh) cohorts in 2 (1.0%); no South Asian cohort was identified. Sixteen studies (7.8%) combined cohorts of different ancestries, including some recent genome-wide studies with independent replication cohorts from different populations, and 13 (6.4%) described their participants only as mixed or did not report ancestry. Population and ancestry descriptors reported by the original studies are presented in Supplementary Material S3.

The phenotypes investigated covered the full spectrum of the predefined eligibility criteria. These included elite athlete status; endurance-, strength-, power-, sprint-, combat-, and intermittent-sport performance; quantitative measures such as VO_2_max/VO_2_peak, muscle strength, power, muscle-fibre characteristics, and training response; and susceptibility to sport-related injury. Although candidate-gene studies represented a substantial proportion of the literature, more recent studies increasingly incorporated genome-wide, sequencing-based, and multi-variant or polygenic approaches.

Detailed characteristics of the 204 included studies are presented in Supplementary Material S3.

### 3.3. Genes and Polymorphisms Identified

Across the 204 included studies, the dataset contained 378 distinct gene/locus–marker entries, encompassing more than 200 genes or genomic loci and including single-nucleotide variants, insertion/deletion polymorphisms, VNTRs, haplotypes, mitochondrial variants, intergenic loci, and other genetic-marker categories.

The most frequently investigated variant was *ACTN3* rs1815739, which was analysed individually in 47 included studies, followed by the *ACE* insertion/deletion polymorphism, analysed individually in 33 studies. Other recurrently investigated markers included *PPARGC1A* rs8192678 (14 studies), *AMPD1* rs17602729 (13 studies), *AGT* rs699 (11 studies), *PPARA* rs4253778 (9 studies), *ADRB2* rs1042713 (9 studies), *NOS3* rs2070744 (9 studies), *SLC16A1* rs1049434 (8 studies), and *PPARG* rs1801282 (7 studies). Furthermore, some recurrent markers were incorporated into total-genotype-score or other multi-variant models without being independently associated with the phenotype under investigation; such multi-variant studies are listed among the Study IDs of Supplementary Material S3 but are not included in the marker counts given above. The identified genes represented a broad range of biological systems relevant to physical performance, including muscle structure and contraction, cardiovascular regulation and blood flow, oxygen transport and hypoxic signalling, energy and substrate metabolism, mitochondrial function, growth and muscle remodelling, inflammatory pathways, neurotransmission, and connective-tissue integrity. More recently identified loci also arose from hypothesis-free genome-wide and sequencing-based studies and therefore extended beyond genes traditionally selected on the basis of known exercise physiology.

To facilitate a design-aware interpretation of the available evidence, the principal gene–variant–phenotype associations and polygenic models discussed in this review were classified according to the predefined three-tier evidence framework. The resulting evidence-strength classifications are summarized in Table 1. The complete gene- and marker-level dataset, including the investigated phenotype and number of studies in which each marker was represented, is provided in Supplementary Material S1

## 4. Discussion

This review provides an updated synthesis of the literature investigating genetic variation in relation to athletic status, physical-performance phenotypes, training response, cardiorespiratory fitness, and sport-related injury. By integrating 204 studies published up to 1 March 2026, the present review captures almost three decades of research spanning traditional candidate-gene studies, genome-wide and sequencing-based approaches, and more recent polygenic and multi-variant models. Rather than supporting the existence of individual “performance genes”, the overall evidence depicts athletic performance as a highly complex phenotype influenced by numerous genetic variants whose effects are generally small, context-dependent, and strongly influenced by non-genetic factors.

Interpretation of the literature requires particular consideration of study design. A substantial proportion of sports-genetics research has relied on candidate-gene approaches involving preselected variants and relatively modest or highly selected athlete samples. Although these studies have generated biologically plausible hypotheses and identified several repeatedly investigated markers, individual findings are frequently heterogeneous and independent replication remains limited. By contrast, genome-wide and sequencing-based studies apply substantially more stringent statistical thresholds and reduce the dependence on prior biological assumptions, although their application to elite-athlete populations remains constrained by the limited number of individuals available for sufficiently powered analyses.

Throughout this discussion, greater interpretative weight is therefore given to associations supported by independent replication, larger or multicohort samples, appropriate correction for multiple testing, or convergent evidence from different methodological approaches. Findings from isolated candidate-gene studies are considered primarily hypothesis-generating unless supported by replication or complementary evidence. Similarly, polygenic and total-genotype-score models are considered separately from individual-variant associations because their predictive performance depends not only on the variants included but also on model derivation, external validation, phenotype definition, and population characteristics.

Population ancestry represents an additional source of heterogeneity. Allele frequencies, linkage disequilibrium patterns, and potentially the magnitude or direction of genetic associations may differ between populations, while much of the available sports-genetics literature remains concentrated in selected European and Asian cohorts. Consequently, associations observed in one population should not automatically be extrapolated to others.

Before delving into the discussion of biological pathways, it is worth highlighting *ACE* and *ACTN3* as the most widely studied genes in the field of genetics and sport.

### 4.1. Cardiovascular and Blood Flow Regulation

Proper regulation of blood flow is essential for the supply of oxygen and nutrients and the removal of metabolites during exercise [17]. Two of the most studied genes in this context are *ACE*, which encodes the angiotensin-converting enzyme, and *NOS3*, which encodes endothelial nitric oxide synthase. The insertion–deletion polymorphism of the angiotensin-encoding gene is possibly the most studied marker in relation to athletic performance [18]. Furthermore, together with the R577X polymorphism of *ACTN3*, it was one of the first to be identified.

ACE is a key component of the renin–angiotensin–aldosterone system (RAAS), which regulates blood pressure, sodium balance, and vascular tone. The I/D polymorphism influences circulating angiotensin II levels and, consequently, vasoconstriction capacity [19]. In the present review, *ACE* I/D was one of the most frequently investigated markers, with studies covering strength/power, endurance, intermittent or combat sports, and injury-related outcomes. The I/D polymorphism has been associated with differences in circulating ACE activity, and early candidate-gene studies led to the hypothesis that the I allele might favour endurance-related phenotypes whereas the D allele might be advantageous in strength- and power-oriented contexts. Particularly informative is the study by Wang et al. in elite swimmers from European-derived and East Asian cohorts. In Caucasian swimmers, the D allele was overrepresented among short- and middle-distance athletes, whereas in East Asian swimmers the I allele was overrepresented among short-distance athletes [20].

Differences in sporting phenotype, ancestry, sample size, training status and study design probably contribute to the heterogeneity observed across studies. This interpretation is also consistent with previous systematic reviews and meta-analyses, which have identified associations with athletic performance while simultaneously reporting substantial between-study heterogeneity [18]. The association between *ACE* I/D and athletic-performance phenotypes is therefore best considered Tier 2 evidence: it has been repeatedly investigated and is supported by independent cohorts and meta-analytic evidence, but substantial heterogeneity remains across sporting phenotypes and population groups. The available evidence does not justify classification as Tier 3 because a consistent, independently replicated genome-wide signal supporting a specific performance-related effect is lacking.

*NOS3* encodes endothelial nitric oxide synthase, which catalyses the conversion of L-arginine to nitric oxide, a potent regulator of vascular tone that also contributes to myocardial oxygen consumption and skeletal muscle metabolism and remodelling [21]. The most frequently studied *NOS3* variants are rs1799983 and rs2070744, both associated with reduced enzyme activity or transcription [22,23,24,25]. As with *ACE*, however, the evidence was heterogeneous across sporting phenotypes and populations.

For rs2070744, Gómez-Gallego et al. reported a higher frequency of the T allele and TT genotype in Spanish elite power athletes than in endurance athletes and non-athletic controls, suggesting a possible association with power-oriented performance [23]. Nevertheless, this finding has not translated into a consistent association across the broader literature identified in the present review, in which several studies of endurance and intermittent-sport populations reported null or differing results. Evidence for *NOS3* rs2070744 and rs1799983 remains predominantly Tier 1, as the reported associations derive mainly from candidate-gene studies and have not shown sufficiently consistent independent replication.

Other components of the RAAS and related systems, such as *AGT*, *AGTR1*, *AGTR2* and *BDKRB2*, have also been explored because of their role in blood pressure regulation, but the available evidence is sparse and inconsistent (Tier 1). Taken together, current data indicate that variants in RAAS- and NO-related genes may modestly influence performance by altering vasodilation and blood pressure regulation, potentially favouring certain disciplines, but their effects appear small and strongly context-dependent.

### 4.2. Muscle Structure and Function

The *ACTN3* rs1815739 (R577X) polymorphism is another of the most studied markers in sports genomics. *ACTN3* encodes α-actinin-3, a structural protein that is predominantly expressed in fast-twitch muscle fibres, where it contributes to Z-line integrity and interacts with multiple proteins involved in cytoskeletal organisation and signalling pathways. The 577X variant results in complete α-actinin-3 deficiency, which can alter muscle contractile properties and fibre type profile [4].

In the present review, *ACTN3* rs1815739 was the most frequently studied marker. The evidence was strongest for strength- and power-related phenotypes, which is consistent with the biological role of α-actinin-3 in fast-twitch muscle fibres. Several studies included in the present review reported a higher frequency of the R allele or RR genotype among sprint/power athletes or associations with muscle-power phenotypes. This pattern is supported by previous meta-analyses. Tharabenjasin et al., for example, reported a modest but significant overall association between the R allele and elite power-athlete status, while Ma et al. similarly found an association between the R allele and power events. Nevertheless, the magnitude of these associations was small and varied according to population, sex, analytical model, and study characteristics [3,4].

Evidence for an endurance-related advantage associated with the X allele is less consistent. Although individual studies have reported associations between the X allele or XX genotype and endurance-related phenotypes, other investigations have failed to replicate these findings. Earlier pooled analyses did not provide consistent support for an endurance advantage of α-actinin-3 deficiency, and more recent evidence continues to indicate substantial heterogeneity across studies [18].

The importance of sporting context and population background is also illustrated by multicohort studies. For example, Eynon et al. reported no consistent association between R577X and team-sport athlete status across three European cohorts. Similarly, in the study of elite swimmers by Wang et al., R577X was not associated with swimmer status in either the European or the East Asian cohorts, whereas *ACE* I/D showed population-specific associations [20]. These findings indicate that even for one of the most extensively studied candidate variants, associations observed in sprint/power athletes do not necessarily generalize to all disciplines with high-intensity or explosive demands [26,27]. Overall, *ACTN3* R577X can be considered one of the better-replicated candidate variants in sports genetics, particularly in relation to sprint- and power-oriented phenotypes.

Other genes related to skeletal-muscle structure and function have received less attention. For example, Lin et al. reported an association between variation in *MYL1*, which encodes a myosin light-chain component expressed in skeletal muscle, and interindividual differences in the response to endurance training. However, evidence for *MYL1* remains limited to a small number of studies and requires independent replication before its contribution to performance-related phenotypes can be established (Tier 1) [28].

Current data, including our findings, support R577X as one of the most researched markers, with the R allele potentially being advantageous in many strength and power contexts by enhancing muscle function during exercise, improving the efficiency of muscle glycogen use, or preserving the percentage of fast fibres, among other reasons [29]. The association between *ACTN3* rs1815739 and sprint/power phenotypes can be considered Tier 2 evidence. Unlike many candidate variants, the R577X polymorphism has been investigated in numerous independent populations and is supported by meta-analytic evidence, although effect sizes are modest and substantial heterogeneity remains.

### 4.3. Adrenergic Regulation

The adrenergic system is essential during exercise. In addition to being key in responding to stressful and alert situations, its activation in response to the most demanding physical demands allows for greater mobilisation of energy substrates, optimising muscle and cardiovascular performance. The present review identified variants in *ADRA2A*, *ADRB1*, *ADRB2*, and *ADRB3*, which encode the α2A-, β1-, β2-, and β3-adrenergic receptors, respectively. These receptors mediate the effects of epinephrine (or adrenaline) and norepinephrine, with *ADRB2* being the most studied in relation to performance.

The rs1042713 (Arg16Gly) variant has produced heterogeneous findings across populations and sporting phenotypes. For example, Wolfarth et al. reported that the Gly16 allele was less frequent among elite Caucasian endurance athletes than among sedentary controls, suggesting a relative advantage associated with the Arg16 allele in that population [30]. More recently, Kim et al. observed a higher frequency of the rs1042713 G allele and GG genotype among Korean elite athletes, with an additional association in intermittent-sport athletes [31]. These findings differ in the putatively favourable allele and illustrate the difficulty of extrapolating associations across populations and sporting contexts. Other investigations, including a study comparing Spanish elite endurance and power athletes, failed to identify significant associations for *ADRB2* variants, further supporting a heterogeneous rather than uniform effect [32].

Evidence for other adrenergic receptors is more limited. Santiago et al. reported that carriers of the minor Arg allele of *ADRB3* rs4994 (Trp64Arg) were more frequent among Spanish elite endurance athletes than among non-athletic controls, whereas no significant differences were observed for the *ADRB1* or *ADRB2* variants evaluated in the same cohort [32]. For *ADRB1*, Sawczuk et al. subsequently reported a higher frequency of the Ser49Gly–Arg389Gly haplotype containing the 49Gly allele among endurance athletes than controls, although evidence for this gene remains sparse and has not been extensively replicated [33]. Similarly, *ADRA2A* rs521674 was associated with combat-athlete status in a Polish cohort, but this finding derives from a broader analysis of neurobehavioural and catecholaminergic pathways and requires independent confirmation [34].

The associations involving *ADRB1*, *ADRB2*, *ADRB3* and *ADRA2A* should currently be regarded predominantly as Tier 1 evidence. Although biologically plausible and supported by individual positive studies, the findings have not shown sufficiently consistent independent replication to justify a higher evidence classification.

### 4.4. Growth and Hormonal Pathways

Growth- and remodelling-related pathways have also been investigated in relation to athletic performance. One of the most relevant is *MSTN* (*GDF8*), which encodes myostatin, a negative regulator of skeletal muscle growth. Several variants in *MSTN* have been examined in performance and training-response studies, particularly rs1805086 (K153R) and rs11333758. However, the evidence differs substantially between these variants.

For rs1805086, Santiago et al. reported differences in vertical-jump performance according to genotype in young non-athletic men, whereas Li et al. subsequently observed greater training-induced increases in muscle thickness among carriers of the KR genotype in a small cohort of Han Chinese men undergoing resistance training. In contrast, other studies included in the present review found no association with endurance-athlete status, elite-athlete status, or other performance-related phenotypes. The low frequency of the minor allele in several populations further complicates interpretation. Accordingly, the evidence supporting rs1805086 as a performance-related marker remains Tier 1 [35,36,37].

The evidence for *MSTN* rs11333758 is somewhat more consistent. Ginevičienė et al. reported an overrepresentation of the deletion genotype among Lithuanian endurance-oriented elite athletes, while Leońska-Duniec et al. subsequently identified an association between the same variant and elite/high-elite athlete status in an independent Polish cohort. The latter study also identified an association involving *ACVR2A* rs3764955, a receptor involved in myostatin signalling. Because the rs11333758 association has been observed in independent populations, it can be considered Tier 2 within the framework used in this review [37,38].

*IGF1* and *IGF1R* were also identified, but the evidence for these genes was scarce. Insulin is a key hormone in glucose metabolism. During sports practice, insulin levels decrease and muscle tissue becomes more susceptible to glucose uptake, in addition to promoting the use of fatty acids and glucose from energy stores during prolonged exertion. *IGF1* rs7136446 showed no association with combat-athlete status in the Brazilian cohort in which it was investigated, whereas Ben-Zaken et al. reported differences in *IGF1R* rs1464430 genotype distribution between endurance and power athletes. In particular, the AA genotype was more frequent among endurance than power athletes, but did not differ from non-athletic controls, and its frequency was lower among elite than national-level power athletes. Because these findings derive from a single candidate-gene study and have not been independently replicated within the evidence included here, the *IGF1R* association remains Tier 1 exploratory evidence [39].

On the other hand, the *VEGFA* (vascular endothelial growth factor A) and *VEGFR2* (also known as *KDR*, kinase insert domain receptor) genes encode an endothelial growth factor and one of its main receptors, respectively. This pathway allows the formation of new blood vessels, especially in tissues subjected to high metabolic demands, providing greater delivery of nutrients and oxygen to these tissues. Ahmetov et al. reported that the *KDR* rs1870377 472Gln allele was more frequent among endurance-oriented athletes and was also associated with endurance-related physiological traits, including muscle-fibre composition and, in a subgroup, relative VO_2_max. Conversely, Guilherme et al. found that the alternative genotype pattern was associated with power-athlete status in a large Brazilian cohort. These findings are biologically compatible with a potential role for angiogenesis-related variation in performance, but they do not constitute direct replication of the same phenotype and effect direction across populations [40,41]. Similarly, *VEGFA* rs2010963 has been associated with endurance-oriented athlete status in an individual candidate-gene analysis and has also appeared in subsequent multi-variant models, but independent evidence for a reproducible single-variant effect remains limited. Therefore, the current evidence for individual *VEGFA* and *KDR* variants should still be regarded predominantly as Tier 1.

Taken together, growth, muscle-remodelling and angiogenic pathways remain relevant biological candidates, but with the exception of the strengthened evidence emerging for *MSTN* rs11333758, most associations in this group have not yet achieved the level of independent replication required for stronger classification.

### 4.5. Injury Susceptibility and Inflammation

Injuries are one of the main concerns for any athlete and one of the main reasons that can prevent professionals from achieving success. Because of this, there are several studies in the literature on specific variants and their possible association with injuries. Genetic variation may contribute to interindividual susceptibility, particularly through pathways involved in extracellular-matrix structure and remodelling, muscle function, vascular regulation, and inflammation. However, the strength of the available genetic evidence differs markedly according to both the injury phenotype and study design.

Among candidate genes, *COL5A1* has accumulated some of the most consistent evidence in relation to tendon and ligament injury. Type V collagen contributes to the regulation of collagen fibril formation and therefore represents a biologically plausible determinant of connective-tissue properties. Meta-analytic evidence has associated *COL5A1* variants, particularly rs12722, with susceptibility to tendon and ligament injuries, although the magnitude and direction of the association vary according to genetic model, injury phenotype, and population [42]. More recently, Brazier et al. identified associations involving *COL5A1* rs12722 and the rs12722–rs3196378 haplotype with tendon rupture, ligament sprain, and overall soft-tissue injury history in elite male rugby athletes [43]. The association between *COL5A1* variation and tendon/ligament injury can therefore be considered Tier 2, reflecting convergent evidence across independent candidate-gene studies and meta-analyses.

Other extracellular-matrix and tissue-remodelling genes have shown less consistent results. *MMP3* rs679620 was associated with hamstring injury risk in elite football players in the prospective study by Larruskain et al., and the same variant was subsequently associated with several soft-tissue injury phenotypes in elite rugby athletes. Importantly, however, the multivariable genetic model developed by Larruskain et al. showed acceptable discrimination in the discovery period but failed to discriminate injury risk in the validation period. Moreover, recent meta-analytic evidence has not demonstrated a significant pooled association between rs679620 and tendon/ligament injury. Consequently, despite repeated positive observations, the evidence for *MMP3* rs679620 is best considered Tier 1 rather than a replicated predictive marker [43,44,45].

Genome-wide studies provide a particularly informative contrast with candidate-gene findings. In a cohort of more than 100,000 individuals, Kim et al. found no genome-wide significant associations for Achilles tendon injury or anterior cruciate ligament rupture and were also unable to replicate most previously reported candidate variants after correction for multiple testing [46]. In contrast, genome-wide significant loci have been reported for other musculoskeletal injury phenotypes, including rs6083471 and rs80351309 for medial collateral ligament injury, rs71404070 for rotator-cuff injury, rs35360670 for de Quervain’s tenosynovitis, and intronic *COA1* variants for patellar tendinopathy [47,48,49,50]. Because these findings reached genome-wide significance but generally lack independent external replication of the same association, they are classified here as Tier 2 evidence rather than Tier 3.

More recent work has started to bridge genome-wide discovery with biologically related intermediate phenotypes. Miyamoto-Mikami et al. identified two genome-wide significant loci associated with passive hamstring muscle stiffness in physically active Japanese participants and subsequently tested the identified variants in a separate sample of Japanese athletes. The stiffness-increasing allele of rs12807854 was associated with a history of muscle-strain injury in the athlete cohort, whereas rs78405694 was not. Although this represents stronger convergent evidence than a single candidate-gene association, the follow-up phenotype differed from the original GWAS phenotype; therefore, it is most appropriately considered Tier 2 rather than Tier 3 evidence [51]. Similarly, the GWAS by Ebert et al. identified several suggestive injury-associated loci in high-performance athletes, including variants near *COL22A1*, *PAPPA2*, *PLXNA2*, and other genes, but the relatively small sample and absence of genome-wide significance for the injury analyses mean that these associations remain Tier 1 [52].

Variants in genes traditionally associated with performance have also been examined in relation to injury or exercise-induced muscle damage. For example, *ACTN3* rs1815739 was associated with differences in injury incidence and anatomical distribution among elite endurance runners, whereas a small experimental study reported genotype-related differences in muscle-damage and inflammatory responses following an acute strength-training session. These findings are physiologically interesting but originate from relatively small, phenotype-specific studies and therefore remain Tier 1 evidence for injury susceptibility [53].

Another aspect to consider is inflammation. Inflammation is a natural immune response involved in many biological processes, from infections and wounds to injuries and physical stress [54]. After exercise, an acute response occurs that allows for proper muscle recovery and adaptation to different stimuli. This mechanism is also essential for injury recovery. However, chronic inflammation is more associated with negative aspects, such as overtraining or muscle catabolism or other ailments such as tendinitis [55]. Inflammatory genes, including *IL1B*, *IL1RN* and *IL6*, were also represented among the included studies. The available evidence suggests possible links with performance, recovery or injury-related phenotypes, but the results remain preliminary and should be interpreted cautiously. The IL-6 gene increases its transcription in skeletal muscle, releasing interleukin-6 in large quantities into the blood as an anti-inflammatory response to exercise [56]. In addition, IL-6 has also been associated with glucose homeostasis during exercise and with the modulation of lipid metabolism, increasing lipolysis and fat oxidation [57]. IL-1α is usually released after cell damage, acting as an alarm signal that initiates immediate local inflammation, while IL-1β amplifies the systemic inflammatory response and induces processes such as fever, endothelial activation, or the production of other cytokines such as IL-6 [58].

Among the markers identified in this review, *IL6* rs1800795 has been associated with power-athlete status in a Spanish cohort, but this association was not reproduced in two subsequent Caucasian cohorts. Thus, despite the well-established physiological role of IL-6 during exercise, the genetic association between rs1800795 and athletic phenotype remains Tier 1 [59,60]. Similarly, Cauci et al. reported an association between the intron-2 VNTR of *IL1RN* and athlete status, but independent evidence remains limited, and this finding should also be considered Tier 1 [61].

Overall, genetic susceptibility to sport-related injury is supported by biologically plausible and increasingly diverse evidence, but the field remains far from individual risk prediction. *COL5A1* represents one of the better-supported candidate loci, while several genome-wide significant signals provide promising newer leads.

### 4.6. Neurological and Psychological Factors

Psychological skills and brain-related processes such as motivation, arousal, stress regulation, pain perception, and motor learning are also key determinants of athletic performance [62]. Compared with genes involved in muscle structure, cardiovascular regulation, or metabolism, however, the genetic basis of these neuropsychological characteristics has received substantially less attention in sports genomics. Among the genes identified in the present review, *FAAH*, *COMT*, *SLC6A2*, and *CNTF* represent some of the most frequently investigated candidates in this context.

*FAAH* encodes fatty acid amide hydrolase, a key enzyme in the degradation of endocannabinoids. The rs324420 (C385A; Pro129Thr) variant has been investigated in several athletic populations because of its potential influence on stress responsiveness, pain perception, reward-related behaviour, and other competition-relevant traits. Associations have been reported in football, hockey, volleyball, and broader athlete cohorts, but the direction and phenotype of the reported effects have not been entirely consistent. For example, Silva et al. reported that the A allele was associated with higher performance levels in elite rink-hockey players and subsequently observed a similar association in volleyball players [63,64]. Nevertheless, the broader literature remains heterogeneous, and the variant has not been consistently replicated across comparable sporting populations [65]. Accordingly, the association between *FAAH* rs324420 and athletic performance should currently be regarded as Tier 1, rather than as an established psychogenetic marker.

*COMT* was also identified because of its role in catecholamine metabolism and stress-related behavioural responses. The functional rs4680 (Val158Met) polymorphism has therefore attracted attention in relation to stress responsiveness, cognition, pain perception, and behavioural traits potentially relevant to competition. Antrobus et al. reported that the Val/Val genotype was more frequent among elite rugby athletes than non-athletic controls, while other studies in endurance and combat-sport populations have produced positive as well as null results [66]. Because the available associations arise predominantly from candidate-gene case–control studies and have not shown consistent replication across comparable phenotypes, evidence for *COMT* rs4680 remains Tier 1 [67].

*SLC6A2* encodes the norepinephrine transporter and is therefore directly involved in regulation of noradrenergic signalling, with potential consequences for arousal, attention, mood, learning, and pain perception. Guilherme et al. reported that the rare rs1805065 T allele was absent among Brazilian power athletes, suggesting a possible disadvantage for power-athlete status [68]. In contrast, Fichna et al., studying a substantially larger Polish cohort, found no overall association with athletic status but observed an overrepresentation of the T allele among male high-elite combat athletes [69]. A separate *SLC6A2* variant, rs2242446, has also been associated with combat-athlete status [34]. These population- and phenotype-specific findings do not constitute consistent replication of the same effect and therefore remain Tier 1 exploratory evidence.

*CNTF* encodes ciliary neurotrophic factor, a neurotrophic cytokine involved in motor-neuron support and neuromuscular function. Walsh et al. reported an association between the *CNTF* rs1800169 (1357 G > A) variant and isometric strength adaptation after resistance training in healthy adults [70]. Because this finding derives from a training-response cohort and has not been consistently replicated for comparable athlete-status or performance phenotypes, the evidence remains Tier 1 and hypothesis-generating rather than a validated performance marker.

In general, most studies on the role of genetics in athletic performance focus on the study of markers associated with ‘purely physiological’ factors, when emotional regulation, stress management, or pain tolerance may be parameters just as important as the rest [71]. Collectively, these findings suggest that neuropsychological and neuromuscular pathways represent plausible but still underdeveloped components of sports genetics. Importantly, none of the associations involving *FAAH*, *COMT*, *SLC6A2*, or *CNTF* currently exceeds Tier 1 evidence.

### 4.7. PPAR Family and Mitochondrial Biogenesis

Peroxisome proliferator-activated receptors (PPARs) and coactivators such as PGC-1α play a key role in regulating fatty acid oxidation, mitochondrial biogenesis, and fibre type composition, making their genes candidates for performance-related traits, especially those related to endurance [72,73]. In this review, several polymorphisms were identified in the PPAR family genes and other regulators of mitochondrial biogenesis.

*PPARA* rs4253778 is among the most frequently investigated variants within this pathway. Several candidate-gene studies have associated the G allele with endurance-oriented phenotypes, whereas the C allele has been reported in relation to power-, strength-, or intermittent-sport characteristics. However, pooled evidence is not entirely consistent. Lopez-Leon et al. reported a higher frequency of the G allele and GG genotype among endurance athletes in a meta-analysis of five studies, whereas a subsequent meta-analysis including seven studies found no significant association between rs4253778 and endurance-athlete status [72,74]. Thus, the *PPARA* rs4253778 literature reaches Tier 2 in terms of evidentiary design, because it includes multiple independent cohorts and meta-analytic evaluation, but the association itself remains heterogeneous and its direction cannot be interpreted as a universally favourable genotype.

Stronger convergent candidate-gene evidence has accumulated for *PPARGC1A* rs8192678 (Gly482Ser), which affects a major transcriptional coactivator involved in mitochondrial biogenesis and oxidative metabolism. Earlier studies frequently associated the Gly allele or Gly/Gly genotype with endurance or elite-athlete status, although null and contrasting findings have also been reported [75]. A recent meta-analysis including 21 studies and 5795 athletes found that the Gly/Gly genotype was more frequent among both endurance and power athletes of Caucasian ancestry [76]. Importantly, this pattern was not reproduced in the same direction in Asian populations: no significant association was observed for Asian endurance athletes, while Gly/Gly was less frequent among Asian power athletes. In Japanese women, Yvert et al. found that the rs8192678 AA (Ser/Ser) genotype was associated with a greater proportion of oxidative MHC-I fibres and a lower proportion of MHC-IIx fibres, whereas no corresponding association was observed in men [77]. This ancestry-dependent pattern supports classification of the rs8192678 association as Tier 2, but also demonstrates why the same allele should not be considered universally advantageous across populations.

Evidence for *PPARD* and *PPARG* remains less developed. Maciejewska-Karlowska et al. identified associations between *PPARD* variants and haplotypes and elite-athlete status in a relatively large Polish cohort, including a marked underrepresentation of the rs2267668/rs2016520/rs1053049 A/C/C haplotype among athletes [78]. However, comparable independent replication has been limited, while several other studies included in the present review reported no association for rs2016520 [79]. Similarly, isolated associations involving *PPARG* rs1801282 have been reported in power- or athlete-status phenotypes, but the broader literature remains inconsistent [41,80,81]. Both *PPARD* and *PPARG* associations should therefore currently be considered Tier 1, despite the physiological relevance of these receptors to substrate metabolism and exercise adaptation.

Beyond the PPAR family, nuclear regulators of mitochondrial biogenesis have also been investigated. Of particular interest is *GABPB1* (NRF-2): Eynon et al. reported that the *GABPB1* rs12594956 AA genotype was overrepresented among Spanish elite endurance athletes compared with both power athletes and controls, and an independent Polish cohort subsequently identified the rs12594956 A allele as a positive predictor of elite-athlete status [81,82]. These observations provide independent candidate-gene replication and therefore support a Tier 2 classification for the association of *GABPB1* rs12594956 with endurance/athlete status.

Direct investigation of mitochondrial DNA has similarly yielded predominantly exploratory findings. Whole-mitochondrial-genome sequencing in elite Japanese athletes identified several nominal differences in mtDNA polymorphisms and haplogroup-related variants between endurance, sprint/power, and control groups, but none of the common variant associations remained significant after correction for multiple comparisons [83]. In a training-response context, Harvey et al. found no association between mtDNA variants and responses to high-intensity interval training, although several nuclear-encoded mitochondrial variants were identified in their relatively small cohort [84]. These findings therefore remain Tier 1.

Overall, the PPAR–PGC-1α–mitochondrial axis remains one of the most biologically compelling pathways in sports genetics, but the genetic evidence is heterogeneous. *PPARGC1A* rs8192678 and *GABPB1* rs12594956 currently show the strongest replicated candidate-gene evidence within this group (Tier 2), whereas *PPARA* rs4253778 has also been extensively investigated and meta-analysed but with conflicting pooled results. Associations involving *PPARD*, *PPARG*, other nuclear regulators of mitochondrial biogenesis, and mtDNA remain primarily Tier 1.

### 4.8. Metabolic Pathways

Energy production, substrate utilization, lactate transport, oxygen sensing, and iron homeostasis are central determinants of the physiological response to exercise. Accordingly, numerous genes involved in these processes have been investigated in relation to athletic status and performance. Among the most frequently studied metabolic candidates in the present review were *AMPD1*, *HIF1A*, *SLC16A1*, *HFE*, and *CKM*, although the consistency and methodological strength of the evidence differed considerably between loci.

*AMPD1* encodes the skeletal-muscle isoform of AMP deaminase, which participates in purine nucleotide metabolism and contributes to the maintenance of adenine nucleotide balance during intense muscular activity [85]. The rs17602729 (C34T) nonsense variant reduces AMPD1 activity and has been widely investigated in both endurance- and power-oriented athletes [86]. A recent meta-analysis including 20 studies and 5717 participants found the CC genotype to be overrepresented in both endurance and power athletes compared with non-athletic controls, with no significant difference in genotype distribution between endurance and power athletes [87]. An earlier meta-analysis focused on power athletes similarly identified the C allele as more frequent among power athletes [88]. Taken together, the association between *AMPD1* rs17602729 and athlete status can therefore be considered Tier 2. However, the available evidence supports a general association with athletic status more clearly than a specific predisposition towards either endurance or power performance.

*HIF1A* encodes the α-subunit of hypoxia-inducible factor 1, a transcription factor involved in cellular adaptation to reduced oxygen availability through regulation of glycolysis, glucose transport, angiogenesis, and other metabolic pathways [89]. The rs11549465 (Pro582Ser) variant has been investigated in relation to strength and power performance [90]. Several candidate-gene studies have reported a greater frequency of the Ser allele among strength/power athletes, although endurance-related findings have been inconsistent [80,91]. Importantly, meta-analytic evidence supports an association between the Ser allele and power-athlete status [88]. Within the present framework, the rs11549465–power phenotype association is therefore classified as Tier 2 evidence.

Lactate transport represents another relevant metabolic mechanism. *SLC16A1*, which encodes monocarboxylate transporter 1 (MCT1), facilitates the transport of lactate and other monocarboxylates across cell membranes and is highly expressed in oxidative skeletal-muscle fibres [92]. Its rs1049434 (T1470A; Glu490Asp) variant has been examined across endurance, power, and combat-sport populations [93]. Guilherme et al., using Brazilian and European cohorts, found the T allele to be overrepresented among endurance athletes and associated the TT genotype with lower exercise-induced blood lactate accumulation and higher VO_2_max [94]. In contrast, a recent study of Japanese long-distance runners reported an increasing frequency of the AA genotype with competitive level and higher aerobic-capacity measures among AA carriers [95]. Thus, the presence of associations in multiple independent populations supports Tier 2 evidence for a relationship between *SLC16A1* rs1049434 and performance-related phenotypes, but the direction of the effect is clearly population- and phenotype-dependent [96].

Iron metabolism may also contribute to endurance-related phenotypes because adequate iron availability is required for oxygen transport and oxidative metabolism. Among the variants examined, *HFE* rs1799945 (H63D) shows comparatively consistent candidate-gene evidence [97]. Semenova et al. reported a higher frequency of the iron-increasing CG/GG genotypes among both Russian and Japanese elite endurance athletes compared with ancestry-matched controls. Their meta-analysis of five cohorts comprising 586 athletes and 1416 controls similarly showed an overrepresentation of CG/GG genotypes among endurance athletes, while the G allele was also associated with higher VO_2_max in a subgroup of Russian male athletes [98]. This combination of replication across populations and meta-analytic support places the *HFE* rs1799945–endurance association within Tier 2.

Another interesting gene that has been studied is the *CKM* gene, which encodes the muscle isoform of creatine kinase. Phosphocreatine stored in muscle tissue acts as an immediate source of energy during physical exertion [99]. Creatine kinase mediates the conversion of phosphocreatine to creatine via ADP, so that ATP can be used during muscle contraction. Although there are studies that identify the role of rs8111989 polymorphism in performance, especially in strength and power sports, only two studies have been identified in our review, with no significant associations [100,101].

Finally, mention should be made of the *CYP1A2* gene, which encodes a hepatic cytochrome P450 enzyme responsible for metabolising caffeine [102]. The rs762551 polymorphism classifies individuals as fast or slow caffeine metabolisers, which influences both the magnitude of the performance-enhancing effect and the likelihood of adverse responses [103,104]. *CYP1A2* rs762551 was identified in relation to caffeine metabolism and exercise response. Guest et al. reported genotype-dependent effects of caffeine on endurance performance, whereas another study included in the present review did not identify a significant genotype interaction [103]. However, subsequent systematic reviews and meta-analyses provide broader evidence that the ergogenic response to caffeine differs according to rs762551 genotype, particularly for endurance-type outcomes [105]. Because this association has been evaluated across multiple intervention studies and meta-analyses, the *CYP1A2* rs762551–caffeine response relationship can be considered Tier 2 evidence.

### 4.9. Genome-Wide and Polygenic Approaches: From Discovery to Prediction

The gradual shift from candidate gene studies to genome-wide discovery and polygenic modelling represents one of the most significant methodological advances in the field. Sporting performance and related physiological traits are highly complex phenotypes that are difficult to explain adequately by a small number of common variants with large individual effects. Genome-wide association studies (GWAS) reduce dependence on prior biological assumptions and apply stringent correction for multiple testing, whereas polygenic approaches attempt to capture the cumulative contribution of multiple variants. However, these approaches also present specific challenges in sports science, particularly the limited availability of large elite-athlete cohorts, phenotype heterogeneity, and the need for independent validation.

Some of the strongest evidence for specific athlete-status loci in the present review derives from multi-stage GWAS. Al-Khelaifi et al. investigated endurance-athlete status in a European discovery cohort followed by replication in independent Russian and Japanese cohorts. Although no variant reached conventional genome-wide significance in the initial discovery analysis, subsequent replication and meta-analysis identified *MYBPC3* rs1052373 at genome-wide significance, with the same direction of association across cohorts and additional associations with VO_2_max and metabolomic phenotypes. Under the evidence framework adopted in this review, this association therefore fulfils the criteria for Tier 3 (higher-strength evidence) because a genome-wide signal was supported across independent populations [106].

More recently, Wang et al. conducted a multi-phase, multi-ethnic GWAS of world-class sprint/power athletes from Jamaican, African-American, and Japanese populations and subsequently tested the leading signal in two European replication cohorts. The G allele of *GALNT13* rs10196189 was consistently associated with sprint/power-athlete status across populations, and the combined analysis exceeded the conventional genome-wide significance threshold [107]. The association was subsequently reproduced in an independent cohort of Han Chinese sprint/power athletes by Chen et al., in whom the G allele was again overrepresented among athletes [108]. This combination of hypothesis-free discovery, cross-ancestry consistency and independent replication makes the *GALNT13* rs10196189 association one of the clearest examples of Tier 3 evidence identified in the present review.

Genome-wide studies of intermediate performance-related phenotypes provide complementary evidence. In the HUNT3 Fitness Study, Klevjer et al. performed a GWAS using directly measured VO_2_peak in 4525 participants. Genome-wide significant associations were identified, including sex-specific signals, and two variants identified in women, rs376927175 near *APBA1* and a PIK3R5-linked signal, showed nominal replication in approximately 60000 UK Biobank participants [109]. Because the replication phenotype was derived using a different CRF assessment and only a subset of the initial signals replicated, these findings are more appropriately considered Tier 2.

Training-response studies further demonstrate why genome-wide design alone does not guarantee a reproducible predictor. Bouchard et al. performed a GWAS of VO_2_max response to standardized endurance training in the HERITAGE Family Study and derived a 21-variant genomic predictor in which individuals at opposite ends of the score distribution differed substantially in their average training response. Several individual loci were subsequently replicated in other exercise-training cohorts, although the complete 21-variant score was not independently validated as a model [110]. A decade later, Williams et al. conducted a multicentre GWAS of VO_2_peak training response in 507 participants and generated a 12-variant polygenic predictor score. No locus reached statistical significance at the genomic level and, more importantly, the predictor failed both internal cross-validation and testing on an independent validation sample [111]. Therefore, variants in the discovery phase that appear promising do not necessarily lead to reproducible polygenic predictions.

These studies also illustrate an important distinction between traditional total genotype scores (TGS) and modern genome-wide polygenic scores (PGS). Most sports-specific TGS models were constructed by selecting a relatively small number of candidate variants previously associated with performance and assigning favourable alleles an unweighted or weighted score. Early examples include the six-variant power-oriented TGS proposed by Ruiz et al. and the power and endurance genetic-distance scores developed by Ben-Zaken et al. Such models were able to differentiate athlete groups at the population level, but were generally developed and tested in relatively small cohorts without independent external validation [112,113]. Consequently, under the present framework, these models remain Tier 1 exploratory evidence, irrespective of the number of variants included.

Subsequent studies have increased model complexity but have not eliminated this limitation. Guilherme and Lancha evaluated power-oriented TGS models containing up to ten variants in Brazilian athletes and found greater scores among power athletes than non-athletes; however, even the best-performing model showed limited sensitivity, illustrating the difference between statistically distinguishing groups and accurately identifying individual athletes [114]. More recently, Wang et al. evaluated 2075 sports-related variants in Chinese sprint/power athletes and constructed a 30-variant model that achieved an area under the receiver-operating-characteristic curve of 0.707 in a held-out validation subset [115]. Although this represents a methodological improvement over simple candidate TGS approaches, the validation sample originated from the same underlying population and the model has not yet been externally validated. It therefore remains Tier 1 exploratory polygenic evidence.

An example of a PGS that extends beyond athlete-status phenotypes is the model developed by Øvretveit et al. They developed a CRF PGS using effect estimates from a GWAS of directly measured CRF in HUNT, model development in 65,165 UK Biobank participants, and subsequent evaluation in more than 82,000 independent HUNT participants who were not part of the discovery sample. Individuals in the highest versus lowest PGS decile differed by approximately 1.55 mL·kg^−1^·min^−1^ in CRF. This demonstrates that genome-wide polygenic information can capture a measurable component of interindividual variation in CRF and represents substantially stronger methodology than traditional small candidate-based TGS models [116]. Under the present framework, this model meets Tier 3 criteria for prediction of cardiorespiratory fitness in population cohorts, while remaining unvalidated for elite-athlete status. Transferability across ancestries also remains a major limitation because allele frequencies and linkage disequilibrium patterns differ between populations; a PGS developed for population-level CRF should therefore not be assumed to have equivalent discriminatory performance in elite athletes or across ancestry groups without direct validation.

Finally, two recent mechanistic genomic studies, which are not among the 204 studies included in this review, illustrate how hypothesis-free discovery may uncover biological pathways that are not readily identified through traditional sport-related search terms. Ström et al. identified the rs115660502 G allele at genome-wide significance for increased capillarisation. The same allele was more frequent in elite endurance athletes than in power athletes and controls in two independent cohorts, and functional experiments implicated *RAB3GAP2* in endothelial-cell proliferation and angiogenesis [117]. Similarly, Nakamichi et al. reported a higher frequency of the gain-of-function *PIEZO1* E756del variant among Jamaican sprinters and demonstrated experimentally that increased *PIEZO1* signalling alters tendon properties and physical performance [118].

Overall, the evidence identified in this review supports a methodological transition from isolated candidate variants towards genome-wide discovery and polygenic modelling. The strongest current genetic associations are those that combine stringent genome-wide thresholds with independent replication, as illustrated by *MYBPC3* rs1052373 and *GALNT13* rs10196189. In contrast, most athlete-specific TGS models remain exploratory because they are based on preselected candidate variants and lack external validation.

### 4.10. Evidence from Markers: Comparison with Other Reviews

The present findings are broadly consistent with previous systematic reviews and meta-analyses that have focused on a smaller number of polymorphisms:

Several systematic reviews and meta-analyses have provided converging evidence on the role of classic polymorphisms in athletic performance. Ma et al. (2013) reported that the *ACE* I/D and *ACTN3* R577X variants are associated with performance and that the *ACE* II genotype and *ACTN3* R allele favour endurance and power tests, respectively [3]. In footballers, McAuley et al. found significant associations of the *ACE* D and *ACTN3* R alleles with player status, consistent with the intermittent but power-oriented nature of the sport [119]. Ipekoglu et al. observed an association between the *ACE* II genotype and endurance performance but found no clear relationship with the *PPARA* rs4253778 polymorphism [74]. Weyerstraß et al. identified several markers with potential relevance to power sports, including the *ACE* D allele, the *ACTN3* R allele, and variants in *AGT*, *IL6*, *NOS3*, and *SOD2* (MnSOD) [120]. More recently, Konopka et al. (2022) reported significant associations between endurance athlete status and the *ACE* II, *ACTN3* XX, *PPARGC1A* rs8192678 GG, *AMPD1* rs17602729 CC, and *HFE* rs1799945 GG/GC genotypes [121]. Taken together, *ACE* I/D remains one of the most extensively investigated variants in sports genetics, but its association with athletic-performance phenotypes is heterogeneous across disciplines and populations, consistent with the Tier 2 classification used in the present review.

In addition to the reviews and meta-analyses mentioned in the previous paragraph, *ACTN3* has further supporting evidence: Tharabenjasin, Pabalan, and Jarjanazi recently published a meta-analysis in which they identified an association between the R allele and power disciplines to a greater or lesser extent depending on gender and ancestry [4]. Taken together, *ACTN3* R577X is one of the better-replicated candidate variants, particularly for sprint/power phenotypes, but effect sizes are modest and substantial heterogeneity remains across sex, ancestry, and sporting context; this is consistent with Tier 2 rather than higher-strength evidence.

Several meta-analyses have further highlighted the potential contribution of metabolic and transcriptional regulators to athletic performance. A systematic review by Petr et al. (2019) on genes encoding peroxisome proliferator-activated receptors (PPARs) included 27 articles and revealed strong associations for *PPARA* rs4253778 (C allele), *PPARGC1A* rs8192678 (Gly allele), and *PPARD* rs2016520 (C allele), which is consistent with our findings on possible performance-related associations for *PPARA* and *PPARGC1A* markers, especially in endurance disciplines [122]. Subsequently, Chen et al. analysed *PPARGC1A* rs8192678 in 10 studies (3708 athletes and 6228 controls) and concluded that the Gly allele may be beneficial in both power and endurance events, the latter mainly in Caucasian populations [123]. Complementing these results, a meta-analysis by Ipekoglu et al. examined polymorphisms in *AGT*, *AMPD1*, *HIF1A*, and *IL6* in 23 studies on power sports and found favourable associations for *AGT* rs699 (Thr allele), *AMPD1* rs17602729 (C allele), *HIF1A* rs11549465 (Ser allele) and *IL6* rs1800795 (G allele in male athletes), patterns that are consistent with the associations observed in the studies included in our review [88].

Although our review identified only two articles on the rs8111989 polymorphism of the *CKM* gene, a 2017 meta-analysis by Chen et al. [124] and another from 2022 by Qin et al., which analysed the same variant under its NcoI restriction-fragment designation [125], found significant associations regarding the status of power athletes.

A recent review by Semenova, Hall, and Ahmetov (2023) summarised the current state of sports genetics and, applying strict inclusion criteria, proposed panels of polymorphisms more closely related to the phenotypes of endurance, power, and strength [7]. For endurance, they highlighted variants in *AMPD1*, *CDKN1A*, *HFE*, *MYBPC3*, *NFIA-AS2*, *PPARA*, and *PPARGC1A*; for power, markers in *ACTN3*, *AMPD1*, *CDKN1A*, *CPNE5*, *GALNTL6*, *IGF2*, *IGSF3*, *NOS3*, and *TRHR*; and for strength, variants in *ACTN3*, the androgen receptor (AR), *LRPPRC*, *MMS22L*, *PHACTR1*, and *PPARG*. Markers for the AR, *CDKN1A*, *CPNE5*, *LRPPRC* or *PHACTR1* genes have not been identified in our review; however, for the rest of the polymorphisms mentioned, at least one article has been identified for each.

### 4.11. Strengths and Limitations

This review provides a broad and updated synthesis of 204 studies spanning almost three decades of sports-genetics research and integrates evidence from candidate-gene studies, training-response and quantitative-performance studies, genome-wide and sequencing-based approaches, sport-related injury studies, and polygenic and total-genotype-score models. The search strategy combined two main databases and predefined eligibility criteria; study selection and data extraction followed PRISMA recommendations, and reporting completeness was assessed using a prespecified STROBE/STREGA-derived checklist. In addition, both candidate-gene and genome-wide approaches were considered, allowing the identification of loci beyond the classic candidates and providing a broader view of the state of knowledge on genetics and athletic performance.

This review also has limitations. Although sport-related injury phenotypes were eligible, the search was not designed to comprehensively capture genetic determinants of musculoskeletal disorders outside a sport-related context. Likewise, supplementation was not a standalone search domain, although genotype–supplement interactions were eligible when they directly affected an exercise- or performance-related outcome. STREGA is a reporting guideline rather than a dedicated risk-of-bias instrument; accordingly, the prespecified STROBE/STREGA-derived threshold used here should be interpreted as an assessment of reporting completeness and not as a formal domain-based risk-of-bias assessment. Studies excluded on the basis of reporting completeness may nonetheless have contained informative data. Substantial heterogeneity also exists across the included studies: athletic status and performance were defined using diverse sporting disciplines, competitive levels, physiological outcomes, training interventions, and injury phenotypes, while study populations differed considerably in sample size, sex, ancestry, and training background. Much of the evidence remains concentrated in European and East Asian cohorts, limiting generalizability and the portability of polygenic models across ancestry groups. Genetic markers related to psychological traits were not comprehensively addressed, even though stress regulation, pain perception, motivation, and motor learning are relevant to elite performance. Therefore, some variants related to nutrigenetics, supplementation response, or psychological factors may still have been missed. Finally, as with any keyword-based strategy, relevant reports may not have been retrieved when their genetic findings were not reflected in the title or abstract, or when they were indexed as basic or mechanistic research.

## 5. Conclusions

This systematic review synthesizes evidence from 204 studies and confirms that athletic performance is a complex, highly polygenic phenotype influenced by multiple biological pathways. *ACE* I/D and *ACTN3* rs1815739 remain the most extensively investigated candidate variants, but their effects are modest, heterogeneous, and dependent on phenotype, population, and study design. The increasing use of GWAS, TGS, and PGS better reflects the polygenic nature of performance, although external validation and transferability across populations remain limited. At present, genetic information should therefore be considered primarily a tool for understanding biological variability in performance rather than for individual talent identification, injury prediction, or training prescription. Future studies should prioritize larger multicentre cohorts, greater ancestral diversity, independent replication, and external validation of polygenic models.

## Figures and Tables

**Table 1:** Design-based evidence-strength classification of selected genetic associations and polygenic models discussed in the review.

Gene/Marker or Model	Main Phenotype	Evidence Tier	Basis for Classification
**Cardiovascular and vascular regulation**			
*ACE* I/D	Endurance, power and athlete status	**Tier 2**	Extensively studied across independent cohorts and meta-analyses; direction and magnitude vary by phenotype and population.
*NOS3* rs2070744/rs1799983	Power, endurance and athlete status	**Tier 1**	Predominantly candidate-gene evidence with inconsistent independent replication.
*VEGFA* rs2010963; *KDR* rs1870377	Endurance/power and angiogenic traits	**Tier 1**	Biologically plausible and supported by individual signals, but no consistent same-variant, same-direction replication.
**Muscle structure, growth and injury**			
*ACTN3* rs1815739	Sprint/power athlete status	**Tier 2**	Multiple independent cohorts and meta-analyses support a modest association; null results remain in some sports and populations.
*MSTN* rs11333758	Athlete status	**Tier 2**	Association reported in independent Lithuanian and Polish cohorts.
*MSTN* rs1805086	Muscle power and training response	**Tier 1**	Small genotype–phenotype/training studies with heterogeneous findings and limited replication.
*IGF1R* rs1464430	Endurance vs. power athlete status	**Tier 1**	Single candidate-gene evidence without independent replication.
*COL5A1* rs12722/rs3196378	Tendon, ligament and soft-tissue injury	**Tier 2**	Independent studies plus meta-analytic evidence support a connective-tissue injury association, although effect estimates vary.
*MMP3* rs679620	Hamstring and soft-tissue injury	**Tier 1**	Repeated nominal associations, but predictive models did not validate robustly and pooled evidence is inconsistent.
*COA1* rs149047058/rs142128304	Patellar tendinopathy	**Tier 2**	Genome-wide significant association; independent external replication remains limited.
**Adrenergic, neurobehavioural and neuromuscular pathways**			
*ADRB1*/*ADRB2*/*ADRB3*/*ADRA2A*	Athlete status and performance	**Tier 1**	Isolated candidate-gene associations, differing favourable alleles and limited consistent replication.
*FAAH* rs324420	Sport performance	**Tier 1**	Positive findings in selected sports, but heterogeneous phenotypes and limited independent replication.
*COMT* rs4680	Athlete status/performance-related traits	**Tier 1**	Candidate-gene findings are biologically plausible but inconsistent across sporting contexts.
*SLC6A2* rs1805065/rs2242446	Athlete status, including combat sports	**Tier 1**	Population- and phenotype-specific signals; rare variants and lack of consistent replication limit inference.
*CNTF* rs1800169	Strength-training response/athlete status	**Tier 1**	Sex- and phenotype-specific training-response signal without consistent replication in athlete-status studies.
**PPAR, mitochondrial and metabolic pathways**			
*PPARA* rs4253778	Endurance/athlete status	**Tier 2**	Multiple independent studies and meta-analyses, but pooled findings are conflicting and population-dependent.
*PPARGC1A* rs8192678	Athlete status and oxidative muscle phenotypes	**Tier 2**	Repeated candidate-gene evidence and meta-analysis; effects differ by ancestry, sex and phenotype.
*GABPB1* rs12594956	Endurance/athlete status	**Tier 2**	Independent candidate-gene replication supports a recurrent association, but genome-wide confirmation is lacking.
*PPARD*/*PPARG* variants	Athlete status/metabolic phenotypes	**Tier 1**	Associations remain limited or inconsistent across independent cohorts.
*AMPD1* rs17602729	Endurance and power athlete status	**Tier 2**	A 20-study meta-analysis supports enrichment of the CC genotype in both endurance and power athletes.
*HIF1A* rs11549465	Power athlete status	**Tier 2**	Repeated candidate-gene findings and meta-analytic support for power-oriented phenotypes.
*SLC16A1* rs1049434	Endurance/performance-related phenotypes	**Tier 2**	Associations reported in independent populations, but the direction of the putatively favourable allele is heterogeneous.
*HFE* rs1799945	Endurance athlete status/aerobic capacity	**Tier 2**	Replication in Russian and Japanese cohorts plus meta-analytic support.
*MTHFR* rs1801131	Sprint/strength athlete status	**Tier 2**	Initial and replication cohorts support the association, although broader independent confirmation is limited.
*CYP1A2* rs762551	Caffeine response during exercise	**Tier 2**	Multiple intervention studies and meta-analyses support genotype-dependent ergogenic response; this is not athlete-status evidence.
**Genome-wide and polygenic evidence**			
*MYBPC3* rs1052373	Endurance athlete status/aerobic capacity	**Tier 3**	GWAS discovery followed by Russian and Japanese replication, with genome-wide significance in the combined analysis.
*GALNT13* rs10196189	Sprint/power athlete status	**Tier 3**	Multi-ethnic GWAS with replication in European cohorts and subsequent replication in Han Chinese athletes.
*APBA1*/*MYH10-PIK3R5* loci	Directly measured VO2peak/CRF	**Tier 2**	Genome-wide significant CRF signals with partial or nominal external replication and some phenotype differences.
Candidate-based TGS/TWGS models	Athlete status, training response and injury	**Tier 1**	Usually based on preselected variants and modest cohorts; external validation is generally absent or limited.
Genome-wide CRF PGS	Cardiorespiratory fitness	Tier 3 *	Developed and evaluated across independent discovery, model-development and target samples. Strong for CRF prediction, but not validated for elite-athlete status.

**Note.** Tier 1 = exploratory evidence (typically a single-cohort candidate-gene, genotype–phenotype, training-response, or non-externally validated multi-variant study); Tier 2 = strengthened evidence (independent replication, consistent multicohort/meta-analytic evidence, or a genome-/exome-wide significant signal without full independent replication); Tier 3 = higher-strength evidence (genome-/exome-wide significant association with independent replication in a comparable phenotype, or an externally validated polygenic model for the target phenotype). Tiers describe the methodological strength of the evidence for the stated association or model, not effect size, causality, or individual predictive utility. * The CRF PGS is Tier 3 for prediction of cardiorespiratory fitness in population cohorts, but it has not been validated for elite-athlete status. The table is intentionally selective; complete variant-level data are provided in Supplementary Material S1 and study-level characteristics in Supplementary Material S3.

## Data Availability

The data supporting the findings of this review are provided within the article and its Supplementary Materials.

## Supplementary Materials

The following supporting information can be downloaded at https://www.mdpi.com/article/10.3390/sports14100461/s1: Supplementary Material S1, detailed list of genes and polymorphisms identified in the review; Supplementary Material S2, complete search strategies, search outputs, screening logs, and Boolean operators; Supplementary Material S3, list of identified genetic markers and characteristics of the studies included in the review; Supplementary Material S4, INPLASY protocol (registration no. INPLASY202630012); Supplementary Material S5, completed PRISMA 2020 checklist.
