# Supplementary material for: From Single Markers to Polygenic Panels: A Systematic Review of Genetics in Athletic Performance
Source: Sports (Basel). 2026 Oct 16;14(10):461. doi: 10.3390/sports14100461 (PMC13709855; doi:10.3390/sports14100461)
Supplement: Supplementary file 1 [file sports-14-00461-s001.zip › New - Supplementary Material S3.pdf]

Supplementary Material S3

A) Genes and genetic variants/markers identified in the review

Gene	Variant / marker	No. studies	Impact	Study ID(s)
ABCB1	rs3789243	1 (GWAS)	NA	S111
ACE	rs4311	1	NA	S129
ACE	rs4363	2	P	S10, S164
ACE	rs4424958	1	NA	S129
ACE	I/D	33	P/E/SUP	S1, S3, S10, S16, S26, S28, S31, S36, S44, S47, S62, S64, S66, S70, S73, S75, S84, S96, S97, S122, S129, S132, S135, S137, S142, S146, S148, S149, S153, S157, S164, S169, S175, S176, S178, S181, S185
ACSL1	rs6552828	1	E	S197
ACSL1	rs116143768	1 (GWAS)	E/GA	S18
ACTN3	rs1791690	1	NA	S84
ACTN3	rs1815739	47	P/E/INJ	S1, S4, S12, S13, S16, S26, S27, S31, S34, S39, S43, S47, S51, S54, S61, S62, S66, S70, S74, S75, S80, S82, S84, S86, S94, S99, S109, S121, S131, S133, S135, S136, S137, S140, S142, S146, S153, S154, S156, S157, S160, S164, S168, S172, S173, S181, S185, S194, S195, S198
ACTN3	rs2275998	1	NA	S84
ACVR1B	rs2854464	2	S	S84, S190
ACVR1B	rs10783486	1	NA	S84
ACVR2A	rs3764955	1	GA	S115
ADAMTS14	rs4747096	1	INJ	S112
ADAMTSL3	rs4842924	1 (GWAS)	S	S37
ADRA2A	rs1800544	1	I/E	S181
ADRA2A	rs521674	1	C	S138
ADRB1	rs1801252	1	E	S161
ADRB1	rs1801253	3	E	S26, S158, S161
ADRB2	rs1042713	9	P/I/E	S26, S103, S138, S143, S144, S158, S170, S171,

Gene	Variant / marker	No. studies	Impact	Study ID(s)
				S181, S192
ADRB2	rs1042714	5	I/E	S26, S138, S143, S144, S158, S181
ADRB3	rs4994	5	P/E	S26, S27, S138, S158, S195
AFG3L2	rs7231304	1	E	S87
AGMAT	rs6429759	1 (GWAS)	E	S6
AGT	rs699	11	P/E	S16, S26, S72, S80, S82, S84, S96, S127, S131, S137, S144, S153, S173, S201
AGTR1	rs5186	1	NA	S96
AGTR2	rs11091046	3	P	S79, S82, S84, S200
ALDH2	rs671	1	S	S101
AMPD1	rs17602729	13	P/E/INJ	S3, S25, S33, S35, S65, S75, S84, S131, S142, S146, S157, S181, S182
ANKK1	rs1800497	1	NA	S9
APBA1	rs376927175	1 (GWAS)	E	S108
APOA1	rs5070	1	NA	S26
APOB	rs5742904	1	NA	S26
APOB	rs12713559	1	NA	S26
APOE	rs7412	2	P	S26, S191
APOE	rs405509	1	NA	S9
APOE	rs429358	2	P	S26, S191
APOE	ε2/ε3/ε4	1	NA	S9
ARK2N	rs6507691	1	S	S30
ATP2A3	rs6502758	1 (GWAS)	P/C	S111
BAHD1	rs3803357	1	E	S27
BDKRB2	rs5810761	4	I/E	S142, S162, S173, S181
BDNF	rs6265	3	P/I	S9, S113, S131
C18orf2	rs7279064	1	NA	S113
C5	kgp11512684	1 (GWAS)	NA	S7
CASP8	rs3834129	1	INJ	S112
CCDC73	rs78405694	1 (GWAS)	INJ	S128
CCL2	rs2857656	1	NA	S112
CERS4	rs7258249	1 (GWAS)	E	S6

Gene	Variant / marker	No. studies	Impact	Study ID(s)
CHRNB3	rs4950	2	E	S137, S138
CHRNB3	rs6474412	1	NA	S138
CKM	rs4884	1	NA	S42
CKM	rs344816	1	NA	S42
CKM	rs432979	1	NA	S42
CKM	rs1133190	1	NA	S42
CKM	rs7260359	1	NA	S42
CKM	rs7260463	1	NA	S42
CKM	rs8111989	3	I/E	S67, S75, S181
CKM	rs10410448	1	NA	S42
CKM	NcoI RFLP 1170 bp/985+185 bp	1	NA	S157
CNTF	rs1800169	2	S	S84, S184
CNTFR	rs41274853	2	P	S93, S127
CNTN5	rs10893471	1 (GWAS)	NA	S111
COA1	rs149047058	1	INJ	S107
COA1	rs142128304	1	INJ	S107
COL12A1	rs970547	1	P/I	S69
COL1A1	rs1107946	2	NA	S22, S155
COL22A1	rs3924862	1 (GWAS)	INJ	S46
COL2A1	rs2070739	1	P	S131
COL3A1	rs1800255	1	NA	S22
COL5A1	rs12722	5	P/E/INJ	S21, S22, S90, S131, S145
COL5A1	rs3196378	3	INJ	S21, S22, S90
COL6A1	rs35796750	1	NA	S84
COLGALT1	rs8090	1	NA	S22
COMT	rs4680	4	I	S8, S9, S138, S179
COMT	rs165599	1	NA	S138
COMT	rs2020917	1	NA	S138
CYFIP1	rs8029108	1 (GWAS)	NA	S7
CYP1A2	rs762551	2	SUP	S78, S174
CYP2D6	rs3892097	1	I/E	S181

Gene	Variant / marker	No. studies	Impact	Study ID(s)
CYP27A1	rs41272687	1	E	S87
CYP3A7	rs11568825	1 (GWAS)	E	S6
CYP3A7	rs45446698	1 (GWAS)	E	S6
DAP	rs267939	1 (GWAS)	INJ	S46
DAP	rs267959	1 (GWAS)	INJ	S46
DAP	rs1080440	1 (GWAS)	INJ	S46
DAP	rs2930047	1 (GWAS)	INJ	S46
DCN	rs516115	1	INJ	S112
DIABLO	rs11061368	1	E	S87
DNAPTP6	rs978656	1	NA	S113
DOK5	rs73142922	1 (GWAS)	INJ	S46
DRD1	rs686	1	NA	S138
DRD1	rs4532	1	NA	S138
DRD1	rs265981	1	NA	S138
DRD2	rs6277	1	NA	S138
DRD2	rs4648317	1	NA	S138
DRD2	rs12364283	1	NA	S138
DRD3	rs2283265	1	NA	S138
DSG1	rs10502567	1 (GWAS)	INJ	S46
DSG2	Arg45Gln	1	NA	S26
DSG2	Arg48His	1	NA	S26
DSG2	Cys506Tyr	1	NA	S26
DSG2	Gly811Cys	1	NA	S26
DSG2	Trp305Ter	1	NA	S26
DSG4	rs73410248	1 (GWAS)	INJ	S46
EDN1	rs5370	1	E	S27
EPAS1	rs895436	1	NA	S183
EPAS1	rs1867782	1	NA	S183
EPAS1	rs1867785	1	P	S183
EPAS1	rs4035887	1	P	S183
EPAS1	rs11689011	1	P	S183

Gene	Variant / marker	No. studies	Impact	Study ID(s)
EPAS1	Haplotype study	1	E	S91
ESRRA	Variants study	1	NA	S88
ESR1	(TA)n	1	NA	S77
FAAH	rs324420	4	GA	S137, S138, S170, S171
FADS1	rs174547	1 (GWAS)	E	S6
FAM185A	rs113400963	1	E	S87
FEV	rs860573	1	GA	S138
FLJ33581	rs6083471	1 (GWAS)	INJ	S150
FOLH1	rs55729124	1 (GWAS)	E	S6
FST	rs1423560	1	NA	S84
FTO	rs9939609	2	Swimming	S53, S204
GABRB3	rs17116985	1	E	S196
GALNT13	rs10196189	3	P/I	S32, S125, S186
GALNTL6	rs558129	2	S/P	S40, S110
GBF1	rs2273555	1	S	S76†
GLDC	rs4742213	1	NA	S87
GNB3	rs5443	2	NA	S26, S198
GNG12	rs28435277	1 (GWAS)	INJ	S46
GRID2	rs17020631	1 (GWAS)	NA	S7
GRIN2B	rs9988991	1 (GWAS)	NA	S111
GRPEL1-FLJ36777	rs80351309	1 (GWAS)	INJ	S150
GSTM1	Present (1) / null (-)	2	I/E	S26, S181
GSTP1	rs1695	2	I/E	S26, S181
GSTP1	rs1138272	1	NA	S26
GSTT1	Present (1) / null (-)	2	I/E	S26, S181
HFE	rs1799945	5	I/E	S75, S92, S157, S166, S181
HFE	rs1800562	1	E	S92, S181
HFE	rs1800730	1	E	S92
HIF1A	rs1951795	1	NA	S41
HIF1A	rs2301113	1	NA	S41
HIF1A	rs11158358	1	NA	S41
HIF1A	rs11549465	5	P/E	S3, S16, S41, S44, S58,

Gene	Variant / marker	No. studies	Impact	Study ID(s)
				S84
HIF1A	rs11549467	1	NA	S41
HIF1A	rs17099207	1	E	S41
HSD17B14	rs7247312	1	NA	S144
HTR1A	rs6295	1	SKILL	S95
HTR1B	rs6296	1	NA	S138
HTR1B	rs130058	1	NA	S138
HTR1B	rs11568817	1	C	S138
HTR1B	rs13212041	1	NA	S138
HTR2A	rs6311	1	NA	S138
HTR2A	rs6313	1	NA	S138
HTR2A	rs927544	1	NA	S138
HTR2A	rs2274639	1	NA	S138
HTR2A	rs2770296	1	NA	S138
HTR2A	rs7997012	1	NA	S138
HTR2A	rs9567746	1	NA	S138
HTR2C	rs518147	1	NA	S138
HTR2C	rs3813929	1	P	S138
HTR6	rs1805054	1	NA	S138
IGF1	rs7136446	1	NA	S84
IGF1R	rs1464430	1	P	S15
IGF2	rs680	1	NA	S144
IGSF3	rs699785	1	P	S85
IL1RN	rs380092	2	GA	S29, S142
IL1B	rs16944	1	NA	S29
IL1B	rs1143634	1	NA	S29
IL6	rs1800795	6	P/I/E	S16, S26, S49, S123, S125, S144, S152, S153
IQCH	rs7170004	1 (GWAS)	NA	S111
KCNA4	rs1323860	1	E	S57
KCNQ1	rs1323860	1	E	S27
KIF6	rs20455	1	P	S191
KLKB1	rs3733402	1 (GWAS)	E	S6

Gene	Variant / marker	No. studies	Impact	Study ID(s)
LOC102724945	kgp5680198	1 (GWAS)	NA	S7
MAOA	rs1137070	1	NA	S138
MAOA	rs1800464	1	NA	S138
MAPT	rs10445337	2	I	S8, S9
MB	rs7293	1	P/E	S146‡
MCT1 (SLC16A1)	rs1049434	8	P/C/E	S55, S80, S82, S83, S84, S100, S147, S163, S165
MCT1 (SLC16A1)	rs4301628	1	E	S120
MCT1 (SLC16A1)	rs10857983	1	E	S120
MCT2 (SLC16A7)	rs3763979	1	E	S120
MCT2 (SLC16A7)	rs3763980	1	E	S120
MCT4 (SLC16A4)	rs11323780	1	E	S120
MDM4	rs35493922	1	P	S98
MIR608	rs1800255	1	NA	S22
MIR608	rs4919510	1	INJ	S21
MLCK	rs28497577	1	I/E	S181
MLCK	rs2700352	1	I/E	S181
MLN	rs12055409	1	S	S76
MMP3	rs522616	1	GA	S38
MMP3	rs591058	1	NA	S22
MMP3	rs650108	1	NA	S22
MMP3	rs679620	1	INJ	S22
MMS22L	rs9320823	1	S	S102
MMS22L	rs35360670	1 (GWAS)	INJ	S104, S105
MSTN	rs1805086	6	S/P	S42, S75, S84, S117, S153, S157, S159
MSTN	rs3791783	1	NA	S42
MSTN	rs7570532	1	NA	S42
MSTN	rs11333758	2	E/GA	S68, S115
MSTN	rs11681628	1	NA	S42
mtDNA	Mitochondrial haplogroups	1	NA	S87
mtDNA	MT-ND1	1	P	S126
mtDNA	MT-RNR1	1	P	S126

Gene	Variant / marker	No. studies	Impact	Study ID(s)
MTG2	rs6062129	1	E	S87
MTG2	rs6121949	1	E	S87
MTHFR	rs1801131	1	P	S202
MTSS1	rs35360670	1 (GWAS)	INJ	S104, S105
MYBPC3	rs1052373	1 (GWAS)	E	S7
MYH10-PIK3R5	rs190675254/ rs551942830	1 (GWAS)	E	S108
MYL1	rs1472955	1	E	S118
MYLIP	rs3757354	1	E	S27
MYOCD	rs16946588	1	E	S27
MYRF	rs149803	1	NA	S139
MYRF	rs174528	1	NA	S139
MYRF	rs198459	1	C/E	S139
MYRF	rs2238001	1	NA	S139
MYRF	rs7943728	1	E	S139
MYRF	rs61747222	1	GA	S139
NAT2	rs1208	1	E	S26
NAT2	rs1041983	1	NA	S26
NAT2	rs1495741	1 (GWAS)	E	S6
NAT2	rs1799929	1	NA	S26
NAT2	rs1799930	1	NA	S26
NAT2	rs1799931	1	NA	S26
NAT2	rs1801279	1	NA	S26
NAT2	rs1801280	1	NA	S26
NAT8	rs1881245	1 (GWAS)	E	S6
NDUFAF7	rs2041840	1	E	S87
NFATC4	rs2229309	1	E	S3
NFIA-AS2	rs1572312	1 (GWAS)	E	S5
NID1	rs4660148	1	NA	S22
NNMT	rs505978	1	NA	S116
NNMT	rs694539	1	NA	S116
NNMT	rs1941399	1	NA	S116

Gene	Variant / marker	No. studies	Impact	Study ID(s)
NNMT	rs1941404	1	NA	S116
NNMT	rs2155806	1	NA	S116
NNMT	rs2244175	1	NA	S116
NNMT	rs2256292	1	E	S116
NNMT	rs2301128	1	NA	S116
NNMT	rs2511153	1	NA	S116
NNMT	rs2847492	1	NA	S116
NNMT	rs2852432	1	NA	S116
NNMT	rs3819100	1	NA	S116
NNMT	rs4646335	1	NA	S116
NNMT	rs4646337	1	NA	S116
NNMT	rs7109984	1	NA	S116
NNMT	rs10891644	1	NA	S116
NNMT	rs11214926	1	NA	S116
NNMT	rs12285641	1	NA	S116
NNMT	rs55675450	1	NA	S116
NOS3	rs1799983	6	P/I/E	S26, S142, S170, S171, S181, S203
NOS3	rs2070744	9	P/I/E	S9, S16, S26, S44, S71, S80, S82, S84, S131, S153, S181, S203
NPY	rs16139	1	NA	S26
NR1H3	rs7120118	1 (GWAS)	E	S7
NR1H3	rs10838681	1 (GWAS)	NA	S7
NR3C1	rs6198	1	NA	S138
NR3C1	rs33388	1	NA	S138
NR3C1	rs2918419	1	NA	S138
NR3C1	rs56149945	1	NA	S138
NR3C1	ER22/23EK	1	S	S180
NR3C2	rs5522	1	NA	S138
NR3C2	rs5534	1	NA	S138
NR3C2	rs2070951	1	E	S138
NRF1	rs6949152	1	E	S199

Gene	Variant / marker	No. studies	Impact	Study ID(s)
NRF1	Variants study	1	NA	S88
NRF2 (GABPA)	rs1015004	1	NA	S88
NRF2 (GABPB1)	rs7181866	3	C/E	S50, S52, S84, S88
NRF2 (GABPB1)	rs8031031	3	C/E	S50, S52, S84
NRF2 (GABPB1)	rs12594956	4	E	S16, S50, S52, S84, S137
NRF2 (GABPB1)	Variants study	1	NA	S88
OGG1	rs1052133	1	NA	S26
OXTR	rs53576	1	NA	S138
PAPPA2	rs11580456	1 (GWAS)	INJ	S46
PAPSS2	rs10887741	1	NA	S113
PLXNA2	rs11799530	1 (GWAS)	INJ	S46
PPARA	rs4253778	9	P/E/GA	S1, S3, S16, S23, S44, S47, S50, S131, S141, S177
PPARD	rs1053049	1	GA	S119
PPARD	rs2016520	6	GA	S3, S16, S47, S50, S84, S119, S170, S171
PPARD	rs2267668	1	GA	S119
PPARG	rs1801282	7	GA	S3, S44, S47, S80, S84, S137, S170, S171
PPARGC1A	rs8192678	14	P/I/E	S3, S16, S47, S50, S66, S75, S80, S82, S84, S137, S143, S157, S170, S171, S177, S181, S195, S199
PPARGC1A	Variants study	1	NA	S88
PPARGC1B	rs7732671	2	NA	S3, S44
PPARGC1B	Variants study	1	NA	S88
PPP3R1	rs3039851	1	NA	S3
PPRC1	Variants study	1	NA	S88
PROX1	rs10494973	1	E	S27
PTK2	rs7460	2	S	S48, S62
PTK2	rs7843014	2	S	S48, S62
PYROXD2	rs7072216	1 (GWAS)	E	S6
RBFA	rs12964779	1	NA	S87
RBFOX1	rs7191721	1 (GWAS)	E	S5
SERPINA6	rs746530	1	NA	S138

Gene	Variant / marker	No. studies	Impact	Study ID(s)
SGPP1	rs17101394	1 (GWAS)	E	S6
SH3GL3	rs2732158	1 (GWAS)	NA	S111
SHMT2	rs73338162	1	E	S87
SIRT1	rs4746720	1	NA	S138
SIRT1	Variants study	1	NA	S88
SLC22A10	rs75859219	1 (GWAS)	E	S6
SLC22A16	rs12210538	1 (GWAS)	E	S6
SLC2A4	rs5418	1	E	S193
SLC6A13	rs11613331	1 (GWAS)	E	S6
SLC6A2	rs5569	1	NA	S138
SLC6A2	rs36020	1	NA	S138
SLC6A2	rs1805065	4	P/C	S56, S81, S84, S117
SLC6A2	rs2242446	1	C	S138
SLC6A3	rs6347	1	P/C	S138
SLCO1B1	rs4363657	1 (GWAS)	E	S6
SND1	rs322820	1	NA	S87
SOD2	rs4880 / rs1799725	2	GA	S26, S14
SOX10	rs139884	1	NA	S139
SOX10	r	1	NA	S139
SPTLC2	rs1063271	1	E	S87
SPTLC3	rs680379	1 (GWAS)	E	S6
SYNE1	rs2635438	1	NA	S56
TFAM	rs1937	3	E	S3, S47, S137
TFAM	rs2306604	1	GA	S137
TFAM	rs16912201	1	NA	S88
TFB1M	Variants study	1	NA	S88
TFB2M	Variants study	1	NA	S88
TGFA	rs958685	1 (GWAS)	S	S37
TIMM23	rs7085433	1	E	S87
TIMP2	rs4789932	1	NA	S22
TMPRSS11E	rs35307342	1 (GWAS)	E	S6

Gene	Variant / marker	No. studies	Impact	Study ID(s)
TNC	rs2104772	1	S	S62
TPH1	rs1800532	1	NA	S138
TPH1	rs7933505	1	NA	S138
TPH2	rs1386494	1	NA	S138
TPH2	rs7305115	1	NA	S138
TRHR	rs7832552	1	NA	S127
TSHR	rs7144481	1 (GWAS)	E	S5
TTN	rs10497520	1	P	S114
UCP2	rs660339	3	P/E	S3, S47, S142
UCP3	rs1800849	1	E	S3
UGT1A10	kgp2861067	2	NA	S6, S7
UNC119B	rs2066938	1 (GWAS)	E	S6
UNC5C	rs265061	1 (GWAS)	P/C	S111
USP10	rs774294	1 (GWAS)	NA	S111
USP10	rs7187994	1 (GWAS)	NA	S111
VDR	rs9729	1	NA	S17
VDR	rs731236	6	S/INJ	S11, S20, S59, S60, S170, S171, S189
VDR	rs1544410	4	S	S11, S20, S59, S60
VDR	rs2228570	5	S/GA	S11, S20, S59, S60, S189
VDR	rs2544410	1	S	S189
VDR	rs3782905	1	NA	S20
VDR	rs4516035	2	S	S17, S20
VDR	rs7136534	1	S	S17
VDR	rs7975232	2	NA	S59, S60
VDR	rs10735810	1	NA	S17
VDR	rs10783215	1	NA	S20
VDR	rs11568820	1	NA	S17
VDR	rs11574024	1	NA	S17
VDR	rs17882106	1	NA	S17
VEGFA	rs699947	2	NA	S3, S22
VEGFA	rs2010963	1	E	S3, S143
VEGFR2	rs1870377	4	E	S2, S22, S80, S82, S84

Gene	Variant / marker	No. studies	Impact	Study ID(s)
VIPR2	rs3757354	1	E	S27
VNN1	rs3798793	1 (GWAS)	E	S6
ZNF295-C2CD2	rs928874	1 (GWAS)	NA	S89
ZNF295-C2CD2	rs1788355	1 (GWAS)	NA	S89
ZNF608	rs4626333	1	S	S76
-	rs202821	1 (GWAS)	NA	S111
-	rs1949886	1 (GWAS)	NA	S7
-	rs4131754	1 (GWAS)	NA	S111
-	rs4655854	1 (GWAS)	NA	S111
-	rs7698692	1 (GWAS)	NA	S111
-	rs7939474	1 (GWAS)	INJ	S128
-	rs7939583	1 (GWAS)	INJ	S128
-	rs10543242	1 (GWAS)	INJ	S128
-	rs11203550	1 (GWAS)	NA	S111
-	rs12479172	1 (GWAS)	NA	S111
-	rs12505795	1 (GWAS)	NA	S111
-	rs12807854	1 (GWAS)	INJ	S128
-	rs61908632	1 (GWAS)	INJ	S128
-	rs71404070	1 (GWAS)	INJ	S151
-	chr21:47156779:D	1 (GWAS)	INJ	S104, S105

Impact abbreviations: NA, no association; GA, general association; S, strength; P, power; E, endurance; I, intermittent; C, combat; SKILL, skill; INJ, injuries; SUP, supplementation.

B) Study reference key and characteristics of included studies

The Study ID shown at the beginning of the first column corresponds to the identifiers used in Part A.

Population descriptors reported by the original authors grouped following the ancestry categories of the GWAS Catalog.

Study	Population	Ancestry	Sample size (Case + Control)	Gender	Sport discipline / phenotype	Polymorphisms / genetic approach
**S1 -** Ahmetov I et al, 2013	Russian	European	457	Mixed	Healthy subjects (power/strength training)	I/D (ACE), rs1815739 (ACTN3), rs4253778 (PPARa)
**S2 -** Ahmetov II et al, 2009	Russian	European	471+603	Mixed	Various sports	rs1870377

Study	Population	Ancestry	Sample size (Case + Control)	Gender	Sport discipline / phenotype	Polymorphisms / genetic approach
					disciplines	(VEGFR2)
**S3 -** Ahmetov II et al, 2009	Russian	European	287+1132	Mixed	Various sports disciplines	I/D (ACE), rs17602729 (AMPD1), rs11549465 (HIF1A), rs2229309 (NFATC4), rs4253778 (PPARa), rs2016520 (PPARd), rs1801282 (PPARg), rs8192678 (PPARGC1A), rs7732671 (PPARGC1B), rs3039851 (PPP3R1), rs2229309 (NFATC4), rs1937 (TFAM), rs660339 (UCP2), rs1800849 (UCP3), rs2010963, rs699947 (VEGF)
**S4 -** Ahmetov II et al, 2010	Russian	European	456+1211	Mixed	Biathlon, cross-country skiing, walking, cycling, rowing, swimming and triathlon.	rs1815739 (ACTN3)
**S5 -** Ahmetov II et al, 2015	Russian & European controls	European	218+192(R) +1367(E)+230(P)	Mixed	Elite endurance athlete status and VO2max	rs1572312 (NFIA-AS2); rs7191721 (RBFOX); rs7144481 (TSHR)
**S6 -** Al-Khelaifi F et al, 2019	Mixed	Not reported/mixed	490 (GWAS)	Mixed	Endurance disciplines	rs6429759 (AGMAT), rs7258249 (CERS4), rs11568825, rs45446698 (CYP3A7); rs174547 (FADS1), rs55729124 (FOLH1), rs3733402 (KLKB1), rs1495741 (NAT2),

Study	Population	Ancestry	Sample size (Case + Control)	Gender	Sport discipline / phenotype	Polymorphisms / genetic approach
						rs1881245 (NAT8), rs7072216 (PYROXD2), rs17101394 (SGPP1), rs11613331 (SLC6A13), rs75859219 (SLC22A10), rs12210538 (SLC22A16), rs4363657 (SLCO1B1), rs680379 (SPTLC3), rs35307342 (TMPRSS11E), kgp2861067 (UGT1A10), rs2066938 (UNC119B), rs3798793 (VNN1)
**S7 -** Al-Khelaifi F et al, 2020	Russian & Japanese	East Asian; European	796+470+406 (GWAS+Replication Study+Control)	Mixed	Various sports disciplines	kgp11512684 (C5), rs8029108 (CYFIP1), rs17020631 (GRID2), kgp5680198 (LOC102724945), rs1052373 (MYBPC3), rs10838681, rs7120118 (NR1H3); kgp2861067 (UGT1A10), rs1949886 (none)
**S8 -** Antrobus MR et al, 2022	Caucasian	European	635+722	Male	Elite rugby athlete status	rs4680 (COMT); rs10445337 (MAPT)
**S9 -** Antrobus MR et al, 2023	Caucasian	European	645+800	Male	Rugby	rs1800497 (ANKK1), rs405509, ε2/ε3/ε4 (APOE); rs6265 (BDNF), rs4680 (COMT), rs10445337 (MAPT), rs2070744 (NOS3)
**S10 -** Ash GI et al, 2011	Ethiopian	African	114+315	Mixed	Sprinters, 5000m, 10km	I/D (ACE), rs4363 -

Study	Population	Ancestry	Sample size (Case + Control)	Gender	Sport discipline / phenotype	Polymorphisms / genetic approach
					and marathon	A22982G (ACE)
**S11 -** Bahat G et al, 2010	Turkish	Greater Middle Eastern	112	Male	Older, healthy and active men (Strength)	rs2228570 (VDR)
**S12 -** Balberova OV et al, 2023	Russian	European	76+50	Male	Cyclists and sprinters	rs1815739 (ACTN3)
**S13 -** Belli T et al, 2017	Not specified	Not reported/mixed	20	Mixed	Ultramarathon	rs1815739 (ACTN3)
**S14 -** Ben-Zaken S et al, 2013	Israeli Caucasians	European	195+240	Mixed	Sprinters 100m and 200m, long jump, 5000m and marathon runners	rs1799725 (SOD)
**S15 -** Ben-Zaken S et al, 2015	Israeli Caucasians	European	159+96	Mixed	Sprinters 100-200m, jumpers, runners >1500m up to half ironman	rs1464430 (IGF-IR)
**S16 -** Ben-Zaken S et al, 2015	Israeli	European		Mixed	Track and field: 100-200 m sprinters/long jumpers vs 5000 m-marathon runners	I/D (ACE); rs1815739 (ACTN3); rs1800795 (IL-6); rs2070744 (NOS3); rs699 (AGT); rs8192678 (PPARGC1A); rs4253778 (PPARa); rs2016520 (PPARd); rs12594956 (NRF2 (GABPB1)); rs11549465 (HIF1A); PGDS/EGDS polygenic scores
**S17 -** Björk A et al, 2019	Caucasian (mainly)	European	2783	Male	Older men<br><br>(Strength)	rs9729, rs10735810, rs17882106, rs4516035, rs7136534, rs11568820, 11574024 (VDR)
**S18 -** Bojarczuk A et al, 2022	Polish	European	126	Female	Exercise-induced fat loss	rs116143768 (ACSL1)
**S19 -**	Caucasian	European	473	Mixed	VO2max	GWAS (no

Study	Population	Ancestry	Sample size (Case + Control)	Gender	Sport discipline / phenotype	Polymorphisms / genetic approach
Bouchard C et al, 2011					trainability after standardized 20-wk endurance training	genome-wide significant marker); 21-SNP predictor panel
**S20 -** Bozsodi A et al, 2016	Caucasian	European	643	Mixed	Healthy children (Strength)	rs2228570, rs731236, rs1544410, rs4516035, rs3782905 rs10783215 (VDR)
**S21 -** Brazier J et al, 2023	Caucasian	European	590+436	Male	Elite rugby athlete status	13-SNP soft-tissue injury TGS; COL5A1 rs12722 + COL5A1 rs3196378 + MIR608 rs4919510 interaction model
**S22 -** Brazier J et al, 2025	European ancestry	European	184	Male	Elite rugby - soft-tissue injury history	rs1107946 (COL1A1); rs1800255 (COL3A1); rs12722, rs3196378 (COL5A1); rs8090 (COLGALT1); rs1800255 (MIR608); rs591058, rs650108, rs679620 (MMP3); rs4660148 (NID1); rs4789932 (TIMP2); rs699947 (VEGF); rs1870377 (VEGFR2 (KDR))
**S23 -** Broos S et al, 2013	Caucasian	European	500	Male	Healthy subjects (strength training)	rs4253778 (PPARa)
**S24 -** Bulgay C et al, 2023	Turkish discovery; Russian/Finnish replication	European; Greater Middle Eastern	80 (discovery) + replication cohorts	Mixed	Track and field / performance-related traits	Exome-wide association study (no exome-wide significant marker)

Study	Population	Ancestry	Sample size (Case + Control)	Gender	Sport discipline / phenotype	Polymorphisms / genetic approach
**S25 -** Bulgay C et al, 2024	Turkish	Greater Middle Eastern	60+20	Mixed	Track and field athletes	rs17602729 (AMPD1)
**S26 -** Buxens A et al, 2011	Spanish	European	100+53	Male	World-class endurance runners/cyclists vs elite power sprinters/jumpers/throwers	I/D (ACE); rs1815739 (ACTN3); rs1801253 (ADRB1); rs1042713, rs1042714 (ADRB2); rs4994 (ADRB3); rs699 (AGT); rs5070 (APOA1); rs5742904, rs12713559 (APOB); rs429358, rs7412 (APOE); Arg45Gln, Arg48His, Trp305Ter, Cys506Tyr, Gly811Cys (DSG2); rs5443 (GNB3); Present (1) / null (-) (GSTM1); rs1695, rs1138272 (GSTP1); Present (1) / null (-) (GSTT1); rs1800795 (IL-6); rs1801279, rs1041983, rs1801280, rs1799929, rs1799930, rs1208, rs1799931 (NAT2); rs1799983, rs2070744 (NOS3); rs16139 (NPY); rs1052133 (OGG1); rs4880 (SOD2)
**S27 -** Bye A et al, 2020	Caucasian	European	3470+718	Mixed	Directly measured VO2max	rs1815739 (ACTN3); rs4994 (ADRB3); rs3803357 (BAHD1); rs5370 (EDN1); rs1323860 (KCNQ1);

Study	Population	Ancestry	Sample size (Case + Control)	Gender	Sport discipline / phenotype	Polymorphisms / genetic approach
						rs3757354 (MYLIP); rs16946588 (MYOCD); rs10494973 (PROX1); rs3757354 (VIPR2)
**S28 -** Cam S et al, 2007	Caucasian Turkish	Greater Middle Eastern	55	Female	Non-elite athletes (endurance training)	I/D (ACE)
**S29 -** Cauci S et al, 2010	Caucasian	European	205+458	Mixed	Various sports disciplines	rs16944, rs1143634 (IL-1B); 86-bp VNTR intron 2 (IL1RN)
**S30 -** Çığırtaş R et al, 2024	Turkish, Russian & Caucasian	European; Greater Middle Eastern	275+202	Mixed	Various strength, power and endurance disciplines	rs6507691 (ARK2N)
**S31 -** Chae JH et al, 2024	Korean	East Asian	45+679	Mixed	Marathon, 10000m, 5000m, and 10~20 km race walk	I/D (ACE), rs1815739 (ACTN3)
**S32 -** Chen L et al, 2025	Han Chinese	East Asian	49+139	Male	Sprinters, throwers and jumpers	rs10196189 (GALNT13)
**S33 -** Cięszczyk P et al, 2011	Polish	European	127+251	Male	Rowing	rs17602729 (AMPD1)
**S34 -** Cięszczyk P et al, 2011	Caucasian	European	158+254	Male	Sprinters, short-distance swimmers and weightlifters	rs1815739 (ACTN3)
**S35 -** Cieszczyk P et al, 2012	Polish	European	158+160	Male	Sprinters, short-distance swimmers and weightlifters	rs17602729 (AMPD1)
**S36 -** Collins M et al, 2004	Caucasian	European	447+199	Male	Ironman & triathlon	I/D (ACE)
**S37 -** Crossland H et al, 2020	British	European	48+48	Male	Lean body mass / handgrip strength	rs4842924 (ADAMTSL3); rs958685 (TGFA)
**S38 -** Deguchi M et al, 2023	Japanese	East Asian	95	Mixed	Healthy subjects (strength training)	rs522616 (MMP3)

Study	Population	Ancestry	Sample size (Case + Control)	Gender	Sport discipline / phenotype	Polymorphisms / genetic approach
**S39 -** Delmonico MJ et al, 2007	Caucasian	European	157	Mixed	Older men & women (Strength)	rs1815739 (ACTN3)
**S40 -** Díaz Ramírez J et al, 2020	Spanish & Russian	European	85	Mixed/Male	Various sports disciplines and physically active men	rs558129 (GALNTL6)
**S41 -** Döring F et al, 2010	Mixed	Not reported/mixed	303+295	Male	Cross-country skiing, biathlon, cycling and long-distance runners	rs11549465, rs1951795, rs11158358, rs2301113, rs11549467, rs17099207 (HIF1A)
**S42 -** Döring F et al, 2011	Caucasian	European	316+304	Male	Cross-country skiing, biathlon, cycling, running and other endurance sports	rs344816, rs10410448, rs432979, rs1133190, rs7260359, rs7260463, rs4884 (CKM); rs1805086, rs3791783, rs11681628, rs7570532 (MSTN (GDF8))
**S43 -** Döring FE et al, 2010	Caucasian	European	305+292	Male	Cross-country skiing, bi-/triathlon, cycling, running and rowing	rs1815739 (ACTN3)
**S44 -** Drozdovska SB et al, 2013	Ukranian	European	140+260	Mixed	Various sports disciplines	I/D (ACE), rs11549465 (HIF1A), rs2070744 (NOS3), rs4253778 (PPARa), rs1801282 (PPARg), rs7732671 (PPARGC1B)
**S45 -** Dzitkowska-Zabielska M et al, 2022	Polish	European	101+41	Male	Elite athlete status	Candidate-gene/WGS study
**S46 -** Ebert JR et al, 2024	Estonian	European	126 (GWAS)	Mixed	Various sports disciplines	rs3924862 (COL22A1), rs267959, rs2930047, rs1080440, rs267939 (DAP), rs73142922 (DOK5),

Study	Population	Ancestry	Sample size (Case + Control)	Gender	Sport discipline / phenotype	Polymorphisms / genetic approach
						rs10502567 (DSG1), rs73410248 (DSG4), rs28435277 (GNG12), rs11580456 (PAPPA2), rs11799530 (PLXNA2)
**S47 -** Egorova ES et al, 2014	Russian	European	193+840	Male	Football	I/D (ACE); rs1815739 (ACTN3); rs4253778 (PPARa); rs2016520 (PPARd); rs1801282 (PPARg); rs8192678 (PPARGC1A); rs1937 (TFAM); rs660339 (UCP2)
**S48 -** Erskine RM et al, 2012	Caucasian	European	51	Male	Healthy subjects (strength training)	rs7460, rs7843014 (PTK2)
**S49 -** Eynon N et al, 2011	Israeli & Spanish	European	155+205	Mixed/Male	Sprinters and long-distance runners	rs1800795 (IL-6)
**S50 -** Eynon N et al, 2011	Israeli Caucasian	European	74(E)+81(P)+240	Mixed	Long-distance runners and sprinters	rs8031031, rs7181866, rs12594956 (NRF2 (GABPB1)); rs4253778 (PPARa); rs2016520 (PPARd); rs8192678 (PPARGC1A)
**S51 -** Eynon N et al, 2012	Spanish Caucasian	European	154(E)+119(P) +343	Male	Various elite endurance and power disciplines	rs1815739 (ACTN3)
**S52 -** Eynon N et al, 2013	Spanish	European	127+110	Male	Runners of various distances, cycling, rowing, sprinters, throwers and jumpers.	rs8031031, rs7181866, rs12594956 (NRF2 (GABPB1))
**S53 -** Eynon N et al, 2013	Spanish, Polish &	European	32(P)+49(E)+60	Mixed	Various power and endurance	rs9939609

Study	Population	Ancestry	Sample size (Case + Control)	Gender	Sport discipline / phenotype	Polymorphisms / genetic approach
	Russian				disciplines	(FTO)
**S54 -** Eynon N et al, 2014	Caucasian	European	888+568	Male	Various sports disciplines	rs1815739 (ACTN3)
**S55 -** Fedotovskaya ON et al, 2014	Russian	European	323+467	Mixed	Various sports disciplines	rs1049434 (MCT1 (SLC16A1))
**S56 -** Fichna JP et al, 2021	Polish	European	890+1008	Mixed	Various sports disciplines	rs1805065 (SLC6A2), rs2635438 (SYNE1)
**S57 -** Flecha-Velazquez K et al, 2019	Hispanic	Hispanic/Latin American	938+938	Male	Marathon	rs1323860 (KCNA4)
**S58 -** Gabbasov RT et al, 2013	Russian	European	208+1413	Mixed	Weightlifting and wrestling	rs11549465 (HIF1A)
**S59 -** Gabdulkayum A et al, 2025	Kazakhstani	Central Asian	58+34	Male	Elite power athletes - vitamin D status	rs731236, rs1544410, rs7975232, rs2228570 (VDR)
**S60 -** Gabdulkayum A et al, 2026	Kazakhstani - Asian	Central Asian	137	Mixed	Various elite sports (injury predisposition)	rs2228570, rs731236, rs1544410, rs7975232 (VDR)
**S61 -** Garatachea N et al, 2014	Spanish	European	100+283	Mixed	Basketball	rs1815739 (ACTN3)
**S62 -** Gasser B et al, 2023	Caucasian	European	43	Mixed	Alpine and Nordic skiing and sub-disciplines like biathlon	I/D (ACE), rs1815739 (ACTN3), rs7460, rs7843014 (PTK2); rs2104772 (TNC)
**S63 -** Ghosh S et al, 2013	Caucasian (HERITAGE Family Study)	European	473	Mixed	Endurance training - VO2max trainability	GWAS gene-level significance screen
**S64 -** Giaccaglia V et al, 2008	Mixed	Not reported/mixed	213	Mixed	Older, obese and sedentary subjects (strength training)	I/D (ACE)
**S65 -** Gineviciene V et al, 2014	Lithuanian Caucasian	European	84(E)+47(SP) +73(M)+260	Mixed	Endurance, sprint/power and mixed	rs17602729 (AMPD1)

Study	Population	Ancestry	Sample size (Case + Control)	Gender	Sport discipline / phenotype	Polymorphisms / genetic approach
					sports	
**S66 -** Gineviciene V et al, 2016	Russian & Lithuanian	European	161+1202	Mixed	Weightlifting, powerlifting, throwers	I/D (ACE), rs1815739 (ACTN3), rs8192678 (PPARGC1A)
**S67 -** Ginevičienė V et al, 2021	Russian & Lithuanian	European	693+500	Mixed	Various sports disciplines	rs8111989 (CKM)
**S68 -** Gineviciene V et al, 2021	Lithuanian Caucasian elite athletes and non-athlete controls	European	44+127	Mixed	Endurance / sprint-power athlete status	rs11333758 (MSTN (GDF8))
**S69 -** Ginevičienė V & Urnikytė A, 2022	Lithuanian	European	293+287	Mixed	Sprinters, jumpers, throwers, football, long-distance and marathon runners and rowers	rs970547 (COL12A1)
**S70 -** Gómez-Gallego F et al, 2009	Spanish	European	46+46	Male	Road cycling	I/D (ACE), rs1815739 (ACTN3)
**S71 -** Gómez-Gallego F et al, 2009	Spanish	European	153+100	Male	Sprinters, jumpers, throwers, long-distance runners and cyclists	rs2070744 (NOS3)
**S72 -** Gomez-Gallego F et al, 2009	Spanish	European	163+119	Male	Long-distance runners and cyclists, sprinters, jumpers and throwers	rs699 (AGT)
**S73 -** González AJ et al, 2006	Spanish	European	63	Male	Middle and long distance running, swimming, cycling and triathlon	I/D (ACE)
**S74 -** Grealy R et al, 2013	Mixed	Not reported/mixed	196	Mixed	Triathlon	rs1815739 (ACTN3)
**S75 -** Grealy R et al, 2015	Mixed	Not reported/mixed	196	Mixed	Triathlon	I/D (ACE), rs1815739 (ACTN3), rs17602729 (AMPD1),

Study	Population	Ancestry	Sample size (Case + Control)	Gender	Sport discipline / phenotype	Polymorphisms / genetic approach
						rs8111989 (CKM), rs1799945 (HFE), rs1805086 (MSTN (GDF8)), rs8192678 (PPARGC1A)
**S76 -** Grishina EE et al, 2019	Russian elite weightlifters; validation in Polish subelite weightlifters	European	53+87+76	Mixed	Weightlifters	rs4626333 (GBF1); rs12055409 (MLN); rs4626333 (ZNF608)
**S77 -** Grundberg E et al, 2005	Mixed	Not reported/mixed	153	Female	Healthy women<br><br>(Strength)	(TA)n (ESR1)
**S78 -** Guest N et al, 2018	Not specified	Not reported/mixed	101	Male	Various sport disciplines	rs762551 (CYP1A2)
**S79 -** Guilherme JPLF et al, 2018	Brazilian	Hispanic/Latin American	624+731	Mixed	Various sport disciplines	rs11091046 (AGTR2)
**S80 -** Guilherme JPLF et al, 2018	Brazilian	Hispanic/Latin American	327 (P)+964 (C)	Mixed	Top-level endurance and power athlete status	rs1815739 (ACTN3); rs699 (AGT); rs1049434 (MCT1 (SLC16A1)); rs2070744 (NOS3); rs1801282 (PPARg); rs8192678 (PPARGC1A); rs1870377 (VEGFR2 (KDR))
**S81 -** Guilherme JPLF et al, 2019	Brazilian	Hispanic/Latin American	630+926	Mixed	Various sport disciplines	rs1805065 (SLC6A2)
**S82 -** Guilherme JPLF & Lancha AH Jr, 2019	Brazilian	Hispanic/Latin American	285+818; replication: 83+818	Mixed	Power sports; replication in basketball, volleyball, tennis and decathlon	rs1815739 (ACTN3); rs699 (AGT); rs11091046 (AGTR2); rs2887 (CNDP1); rs2346061 (CNDP1/CNDP2); rs3764509 (CNDP2); rs1049434

Study	Population	Ancestry	Sample size (Case + Control)	Gender	Sport discipline / phenotype	Polymorphisms / genetic approach
						(MCT1 (SLC16A1)); rs2070744 (NOS3); rs8192678 (PPARGC1A); rs1870377 (VEGFR2 (KDR)); 10-SNP TGS
**S83 -** Guilherme JPLF et al, 2021	Brazilian, Russian & Hungarian	European; Hispanic/Latin American	633+1442	Mixed	Various endurance disciplines	rs1049434 (MCT1 (SLC16A1))
**S84 -** Guilherme JPLF et al, 2021	Brazilian	Hispanic/Latin American	164+964	Mixed	Various combat disciplines	I/D (ACE), rs1815739 (ACTN3), rs10783486, rs2854464 (ACVR1B); rs699 (AGT), rs11091046 (AGTR2), rs17602729 (AMPD1), rs1800169 (CNTF), rs35796750 (COL6A1), rs1423560 (FST), rs11549465 (HIF1A), rs7136446 (IGF1), rs1049434 (MCT1 (SLC16A1)), rs1805086 (MSTN), rs2070744 (NOS3), rs8031031, rs7181866, rs12594956 (NRF2 (GABPB1)); rs2016520 (PPARd), rs1801282 (PPARg), rs8192678 (PPARGC1A), rs1805065 (SLC6A2), rs1870377 (VEGFR2)
**S85 -**	Russian	European	137+126	Mixed	Sprint/power	rs699785

Study	Population	Ancestry	Sample size (Case + Control)	Gender	Sport discipline / phenotype	Polymorphisms / genetic approach
Guilherme JPLF et al, 2022					athlete status	(IGSF3)
**S86 -** Gutiérrez-Hellín J et al, 2021	Spanish	European	89	Mixed	Runners from 800m to marathon	rs1815739 (ACTN3)
**S87 -** Harvey N.R. et al, 2020	Caucasian	European	62	Mixed	Moderately trained adults (HIIT)	rs7231304 (AFG3L2), rs41272687 (CYP27A1), rs11061368 (DIABLO), rs113400963 (FAM185A), rs4742213 (GLDC), Mitochondrial haplogroups (mtDNA), rs6062129, rs6121949 (MTG2); rs2041840 (NDUFAF7), rs12964779 (RBFA), rs73338162 (SHMT2), rs322820 (SND1), rs1063271 (SPTLC2), rs7085433 (TIMM23)
**S88 -** He ZH et al, 2015	Chinese, Spanish & Polish	East Asian; European	619+1054	Mixed	Long distance and marathon runners	rs2079786, rs2276014, rs731703, rs493052 (ERRα); several NRF1 variants; rs1015004 (NRF2 (GABPA)), rs7181866, rs3784296, rs1797311 (NRF2 (GABPB1)); several, PPARGC1A variants, several PPARGC1B variants, rs2815401, rs4919613 (PPRC1); rs12778366,

Study	Population	Ancestry	Sample size (Case + Control)	Gender	Sport discipline / phenotype	Polymorphisms / genetic approach
						rs11596401 (SIRT1), rs16912201 (TFAM), several TFB1M variants, rs7536768, rs6701836 (TFB2M)
**S89 -** Heckerman D et al, 2017	Italian	European	286+184 (>80)+871 (65-80)	Mixed	Older adults / physical performance	rs928874, rs1788355 (ZNF295-C2CD2)
**S90 -** Heffernan SM et al, 2017	Caucasian	European	540+565	Male	Rugby	rs12722, rs3196378 (COL5A1)
**S91 -** Henderson J et al, 2005	Caucasian	European	492+444	Mixed	Various sports disciplines	Haplotype study (EPAS1)
**S92 -** Hermine O et al, 2015	French	European	129+260	Mixed	Nordic skiing, rowing, judo, petanque	rs1799945, rs1800562, rs1800730 (HFE)
**S93 -** Homma H et al, 2022	Japanese	East Asian	67+416	Mixed	International-level weightlifting status and Wilks performance	rs41274853 (CNTFR); 12-polymorphism power-oriented model and 6-SNP weightlifting-related model
**S94 -** Hong SS & Jin HJ, 2013	Korean	East Asian	150+361	Mixed	Various sports disciplines	rs1815739 (ACTN3)
**S95 -** Humińska-Lisowska K et al, 2024	Polish	European	250+209	Male	MMA, judo, karate, ju-jitsu, boxing and kickboxing	rs6295 (HTR1A)
**S96 -** Karjalainen J et al, 1999	Caucasian	European	80	Mixed	Long-distance runners, orienteers and cross-country skiers and triathlonists	I/D (ACE); rs699 (AGT); rs5186 (AGTR1 (AT1))
**S97 -** Kasikcioglu E et al, 2004	Caucasian	European	29+51	Male	Wrestlers	I/D (ACE)
**S98 -** Kazan HH et al, 2025	Turkish & Russian	European; Greater Middle Eastern	537+763	Mixed	Various power and endurance sports	rs35493922 (MDM4)
**S99 -** Kikuchi N et al, 2014	Japanese	East Asian	253	Mixed	Various power and intermittent	rs1815739

Study	Population	Ancestry	Sample size (Case + Control)	Gender	Sport discipline / phenotype	Polymorphism s / genetic approach
					disciplines	(ACTN3)
**S100 -** Kikuchi N et al, 2017	Japanese	East Asian	199+649	Male	Wrestling	rs1049434 (MCT1 (SLC16A1))
**S101 -** Kikuchi N et al, 2022	Japanese	East Asian	1714+1341	Mixed	Various sports disciplines	rs671 (ALDH2)
**S102 -** Kikuchi N et al, 2022	Russian	European	100+99	Mixed	Weightlifting	rs9320823 (MMS22L); Genotype-phenotype study of 28 previously reported strength markers
**S103 -** Kim MS et al, 2024	Korean	East Asian	226+406	Mixed	Various sports disciplines	rs1042713 (ADRB2)
**S104 -** Kim SK et al, 2017	European	European	5148+97831 / 598+98744	Mixed	Sports injuries	chr21:47156779 :D (intergenic; near COL18A1/SLC19A1/PCBP3); GWAS (no genome-wide significant marker)
**S105 -** Kim SK et al, 2017	European (mainly)	European	3709+88135	Mixed	Sports injuries	rs35360670 (MMS22L); rs35360670 (MTSS1)
**S106 -** Kim SK et al, 2017	European	European	5148+97831 / 598+98744	Mixed	Achilles tendon injury and ACL rupture	GWAS (no genome-wide significant marker)
**S107 -** Kim SK et al, 2021	European ancestry	European	1670+293866	Mixed	Patellar tendinopathy	rs149047058, rs142128304 (COA1)
**S108 -** Klevjer M et al, 2022	HUNT3 Fitness Study	European		Female	Directly measured VO2peak	rs376927175 (APBA1); rs190675254/rs551942830 (MYH10-PIK3R5)
**S109 -** Kobayashi T et al, 2022	Japanese	East Asian	260+26	Female	Various sport disciplines	rs1815739 (ACTN3)
**S110 -** Kozuma A et al, 2025	Japanese	East Asian	723+1139	Mixed	Wrestlers, weightlifters and powerlifters	rs558129 (GALNTL6)
**S111 -**	Turkish,	East Asian;	167+616 (GWAS)	Mixed	Wrestling	rs3789243

Study	Population	Ancestry	Sample size (Case + Control)	Gender	Sport discipline / phenotype	Polymorphisms / genetic approach
Kozuma A et al, 2025	Russian & Japanese	European; Greater Middle Eastern				(ABCB1), rs6502758 (ATP2A3), rs10893471 (CNTN5), rs9988991 (GRIN2B), rs7170004 (IQCH), rs2732158 (SH3GL3), rs265061 (UNC5C), rs7187994, rs774294 (USP10); rs4655854, rs12479172, rs7698692, rs12505795, rs202821, rs11203550, rs4131754 (none)
**S112 -** Larruskain J et al, 2018	Spanish	European	107	Male	Football	rs4747096 (ADAMTS14), rs3834129 (CASP8), rs2857656 (CCL2), rs516115 (DCN)
**S113 -** Lee HH et al, 2021	Mixed	Not reported/mixed	2675	Mixed	Change in physical activity and cardiorespiratory fitness	rs6265 (BDNF); rs7279064 (C18orf2); rs978656 (DNAPTP6); rs10887741 (PAPSS2)
**S114 -** Leonska-Duniec A et al, 2022	Polish sprint/power athletes vs non-athlete controls	European	100+403	Mixed	Sprint/power sports	rs10497520 (TTN)
**S115 -** Leońska-Duniec A et al, 2023	Polish	European	330+365	Mixed	Various sports disciplines	rs3764955 (ACVR2A), rs11333758 (MSTN)
**S116 -** Li JH et al, 2017	Han Chinese	East Asian	498+502 (different modalities)	Male	Healthy subjects	Several NNMT variants
**S117 -** Li X et al, 2014	Han Chinese	East Asian	94	Male	Healthy subjects (strength training)	rs1805086 (MSTN), rs1805065 (SLC6A2)
**S118 -** Lin X et	Han Chinese	East Asian	31+206+99	Mixed+Mal	Various	rs1472955

Study	Population	Ancestry	Sample size (Case + Control)	Gender	Sport discipline / phenotype	Polymorphisms / genetic approach
al, 2017			(case+control+training programme)	e	endurance disciplines	(MYL1)
**S119 -** Maciejewska-Karlowska A et al, 2014	Polish	European	660+704	Mixed	Various sport disciplines	rs2016520, rs2267668, rs1053049 (PPARd)
**S120 -** Maculewicz E et al, 2025	Polish & Czech	European	192+145	Male	Sprinters	rs4301628, rs10857983 (MCT1 (SLC16A1)); rs3763979, rs3763980 (MCT2 (SLC16A7)), rs11323780 (MCT4 (SLC16A4))
**S121 -** Massidda M et al, 2015	Italian	European	178+190	Male	Various sport disciplines	rs1815739 (ACTN3)
**S122 -** Massidda M et al, 2020	Italian & Japanese	East Asian; European	191+519	Male	Football	I/D (ACE)
**S123 -** McAuley ABT et al, 2022	English	European	74+92	Male	Youth vs professional development phase football	rs1800795 (IL-6); 33-SNV TGS/TWGS polygenic profile
**S124 -** McAuley ABT et al, 2023	English & European	European		Male	U12-16 vs U17-23 academy football players	39-SNP maturity-associated polygenic profile (TGS/TWGS)
**S125 -** McAuley ABT et al, 2024	English	European	149	Male	Academy football performance	rs10196189 (GALNT13); rs1800795 (IL-6); 22-SNP polygenic profile associated with power-oriented performance
**S126 -** Mikami E et al, 2013	Japanese	East Asian	100(EMA) +85(SPA)+672	Mixed	Various elite sports; EMA and SPA	MT-RNR1, MT-ND1 (mtDNA)
**S127 -** Miyamoto-Mikami E et al, 2017	Japanese	East Asian	62+649	Mixed	Sprint/power track and field athlete status	rs699 (AGT); rs41274853 (CNTFR); rs7832552 (TRHR)
**S128 -** Miyamoto-Mikami E et al,	Japanese	East Asian	350+1428	Mixed	Passive hamstring stiffness /	rs78405694 (CCDC73); rs12807854,

Study	Population	Ancestry	Sample size (Case + Control)	Gender	Sport discipline / phenotype	Polymorphisms / genetic approach
2025					muscle strain injury	rs61908632, rs7939583, rs10543242, rs7939474 (none)
**S129 -** Moran CN et al, 2006	Greek	European	1027	Mixed	Handgrip strength, vertical jump and other physical-performance tests	I/D, rs4424958, rs4311 (ACE)
**S130 -** Moreland E et al, 2022	Russian	European		Mixed	Weightlifting / powerlifting	28-SNP polygenic strength profile
**S131 -** Murtagh CF et al, 2020	Caucasian	European	535+151	Male	Football	rs1815739 (ACTN3); rs699 (AGT); rs17602729 (AMPD1); rs6265 (BDNF); rs2070739 (COL2A1); rs12722 (COL5A1); rs2070744 (NOS3); rs4253778 (PPARa)
**S132 -** Orysiak J et al, 2013	Caucasian	European	66	Mixed	Cross-country skiing, biathlon and Nordic combined skiing	I/D (ACE)
**S133 -** Orysiak J et al, 2014	Polish	European	200	Male	Canoe, ice hockey, swimming & volleyball	rs1815739 (ACTN3)
**S134 -** Øvretveit K et al, 2025	Norwegian HUNT; UK Biobank validation	European	4,525 HUNT discovery + 65,165 UK Biobank PGS development + 82,109 HUNT target cohort	Mixed	Directly measured cardiorespiratory fitness / VO2peak	Genome-wide polygenic score for cardiorespiratory fitness (CRFPGS)
**S135 -** Papadimitriou ID et al, 2016	Caucasian and African ancestry	African; European	62 / 79	Male	Elite 100-m, 200-m and 400-m sprint performance	I/D (ACE); rs1815739 (ACTN3)
**S136 -** Pasqua LA et al, 2016	Brazilian	Hispanic/Latin American	150	Male	Healthy subjects (endurance training)	rs1815739 (ACTN3)
**S137 -** Peplonska B	Polish	European	413+451	Mixed	Various power and endurance	I/D (ACE), rs1815739

Study	Population	Ancestry	Sample size (Case + Control)	Gender	Sport discipline / phenotype	Polymorphisms / genetic approach
et al, 2017					disciplines	(ACTN3), rs699 (AGT), rs4950 (CHRNB3), rs324420 (FAAH), rs12594956 (NRF2 (GABPB1)), rs1801282 (PPARg), rs8192678 (PPARGC1A), rs1937, rs2306604 (TFAM)
**S138 -** Peplonska B et al, 2019	Polish	European	621+672	Mixed	Various sport disciplines	rs521674 (ADRA2A), rs1042713, rs1042714 (ADRB2); rs4994 (ADRB3), rs4950, rs6474412 (CHRNB3); rs4680, rs2020917, rs165599 (COMT); rs265981, rs4532, rs686 (DRD1); rs6277, rs4648317, rs12364283 (DRD2); rs2283265 (DRD3), rs324420 (FAAH), rs860573 (FEV), rs130058, rs6296, rs11568817, rs13212041 (HTR1B); rs7997012, rs2770296, rs9567746, rs6313, rs2274639, rs927544, rs6311 (HTR2A); rs518147, rs3813929 (HTR2C); rs1805054 (HTR6), rs1137070, rs1800464

Study	Population	Ancestry	Sample size (Case + Control)	Gender	Sport discipline / phenotype	Polymorphisms / genetic approach
						(MAOA); rs56149945, rs33388, rs2918419, rs6198 (NR3C1); rs5522, rs2070951, rs5534 (NR3C2); rs53576 (OXTR); rs746530 (SERPINA6), rs4746720 (SIRT1), rs2242446, rs5569, rs36020 (SLC6A2); rs6347 (SLC6A3), rs7933505, rs1800532 (TPH1); rs7305115, rs1386494 (TPH2)
**S139 -** Pepłońska B et al, 2021	Polish	European	247+281	Mixed	Various sport disciplines	rs61747222, rs174528, rs198459, rs2238001, rs7943728, rs149803 (MYRF); rs139884, rs149435516 (SOX10)
**S140 -** Pereira MA et al, 2022	Brazilian	Hispanic/Latin American		Male	Strength/Injuries (Muscle damage)	rs1815739 (ACTN3)
**S141 -** Petr M et al, 2014	Caucasian	European	77	Male	Ice Hockey (Power measurements)	rs4253778 (PPARa)
**S142 -** Petr M et al, 2022	Caucasian	European	99+107	Male	Football	I/D (ACE), rs1815739 (ACTN3), rs17602729 (AMPD1), rs5810761 (BDKRB2), 86-bp VNTR intron 2 (IL1RN), rs1799983 (NOS3), rs660339 (UCP2)

Study	Population	Ancestry	Sample size (Case + Control)	Gender	Sport discipline / phenotype	Polymorphisms / genetic approach
**S143 -** Pickering C et al, 2018	United Kingdom	European	42	Male	Soccer; 8-week sport-specific aerobic training; Yo-Yo IR1	rs2010963 (VEGF); rs1042713, rs1042714 (ADRB2); rs1205 (CRP); rs8192678 (PPARGC1A); 5-SNP weighted TGS
**S144 -** Pickering C et al, 2019	British	European	48	Not specified	Sprint performance / football player status	rs1042713, rs1042714 (ADRB2); rs699 (AGT); rs7247312 (HSD17B14); rs680 (IGF2); rs1800795 (IL-6)
**S145 -** Posthumus M et al, 2011	Caucasian	European	313	Male	Triathlon	rs12722 (COL5A1)
**S146 -** Pranckevicien e E et al, 2021	Lithuanian	European	180+255	Mixed	Endurance, sprint/power and mixed sports	Four-marker endurance-mixed-power TGS (ACE, ACTN3, AMPD1 and MB); individual genotype/allele comparisons also performed
**S147 -** Ramírez de la Piscina-Viúdez X et al, 2021	Spanish	European	85+107	Mixed	Triathlon	rs1049434 (MCT1 (SLC16A1))
**S148 -** Rankinen T et al, 2000	Canadian, German, Finnish & American	European	192+189	Male	Biathlon, cross-country skiing, long-distance running, road cycling & Nordic combined	I/D (ACE)
**S149 -** Rodríguez-Romo G et al, 2010	Spanish	European	281	Mixed	Healthy subjects (power training)	I/D (ACE)
**S150 -** Roos AK et al, 2017	European	European	1,564 genotyped+100,5 44 genotyped	Mixed	Sports injuries	rs6083471 (FLJ33581); rs80351309 (GRPEL1-FLJ36777)
**S151 -** Roos TR et al, 2017	European	European	8,159 genotyped+92,44	Mixed	Sports injuries	rs71404070 (none)

Study	Population	Ancestry	Sample size (Case + Control)	Gender	Sport discipline / phenotype	Polymorphisms / genetic approach
			4 genotyped			
**S152 -** Ruiz JR et al, 2010	Spanish	European	153+100	Male	Sprinters, throwers, jumpers, long-distance runners and road cyclists	rs1800795 (IL-6)
**S153 -** Ruiz JR et al, 2010	Spanish Caucasian	European	53(P)+100(E) +100(C)	Male	Elite sprinters/jumpers vs distance runners/road cyclists and nonathletic controls	I/D (ACE); rs1815739 (ACTN3); rs699 (AGT); rs1805086 (MSTN (GDF8)); rs1800795 (IL-6); rs2070744 (NOS3); 6-SNP power TGS
**S154 -** Ruiz JR et al, 2011	Spanish	European	66+334	Mixed	Volleyball	rs1815739 (ACTN3)
**S155 -** Saito M et al, 2022	Japanese, Polish & Russian	East Asian; European	218+1929	Mixed	Climbers and Healthy Subjects	rs1107946 (COL1A1)
**S156 -** Santiago C et al, 2010	Spanish	European	283	Mixed	Healthy subjects (power training)	rs1815739 (ACTN3)
**S157 -** Santiago C et al, 2010	Spanish	European	39+15	Male	Lightweight rowing	I/D (ACE); rs1815739 (ACTN3); rs17602729 (AMPD1); NcoI RFLP 1170 bp/985+185 bp (CKM); rs1799945 (HFE); rs1805086 (MSTN (GDF8)); rs8192678 (PPARGC1A)
**S158 -** Santiago C et al, 2011	Spanish	European	153+100	Male	Long-distance cyclists, throwers, sprinters and jumpers	rs1801253 (ADRB1), rs1042713, rs1042714 (ADRB2); rs4994 (ADRB3)
**S159 -** Santiago C et al, 2011	Spanish	European	216	Male	Healthy subjects (Power measures)	rs1805086 (MSTN (GDF8))

Study	Population	Ancestry	Sample size (Case + Control)	Gender	Sport discipline / phenotype	Polymorphisms / genetic approach
**S160 -** Saunders CJ et al, 2007	Caucasian	European	457+143	Male	Triathlon	rs1815739 (ACTN3)
**S161 -** Sawczuk M et al, 2012	Polish	European	223+354	Mixed	Various power & endurance disciplines	rs1801253, rs1801252 (ADRB1)
**S162 -** Sawczuk M et al, 2013	Polish & Russian	European	1124+1191	Mixed	Various sports disciplines	rs5810761 (BDKRB2)
**S163 -** Sawczuk M et al, 2015	Polish	European	212+621	Mixed	Various sports disciplines	rs1049434 (MCT1 (SLC16A1))
**S164 -** Scott RA et al, 2010	Jamaican	African	114+311	Mixed	100-400 m sprint, jumps and throws	I/D, rs4363 (ACE); rs1815739 (ACTN3)
**S165 -** Seki S et al, 2024	Japanese	East Asian	158+649	Male	Long-distance runners	rs1049434 (MCT1 (SLC16A1))
**S166 -** Semenova EA et al, 2020	Russian & Japanese	East Asian; European	315+809	Mixed	Biathlon, kayaking, cross-country skiing, cycling, rowing, running >400m, swimming >400m and triathlon.	rs1799945 - H63D (HFE)
**S167 -** Semenova EA et al, 2022	Russian, Finnish & Japanese	East Asian; European	645+333 (GWAS+replication)	Mixed	Muscle-fiber composition and endurance vs power athlete status	GWAS of muscle-fiber composition
**S168 -** Shang X et al, 2010	Han Chinese	East Asian	250+450	Mixed	Rowing, runners >5000m, marathon, long-distance cycling, swimming >400m	rs1815739 (ACTN3)
**S169 -** Shuler K et al, 2019	Not specified	Not reported/mixed	88	Mixed	Older subjects<br><br>(Strength)	I/D (ACE)
**S170 -** Silva HH et al, 2022	Caucasian (mainly)	European	116	Mixed	Hockey	rs1042713 (ADRB2), rs324420 (FAAH), rs1799983 (NOS3), rs2016520 (PPARd), rs1801282 (PPARg),

Study	Population	Ancestry	Sample size (Case + Control)	Gender	Sport discipline / phenotype	Polymorphisms / genetic approach
						rs8192678 (PPARGC1A), rs731236 (VDR)
**S171 -** Silva HH et al, 2023	Caucasian (mainly)	European	228	Mixed	Volleyball	rs1042713 (ADRB2), rs324420 (FAAH), rs1799983 (NOS3), rs2016520 (PPARd), rs1801282 (PPARg), rs8192678 (PPARGC1A), rs731236 (VDR)
**S172 -** Silva MS et al, 2015	Brazilian	Hispanic/Latin American	206	Male	Healthy subjects (endurance training)	rs1815739 (ACTN3)
**S173 -** Silvino VO et al, 2025	Brazilian	Hispanic/Latin American	91+74	Male	Handball	rs1815739 (ACTN3), rs699 (AGT), rs5810761 (BDKRB2)
**S174 -** Spineli H et al, 2020	Not specified	Not reported/mixed	101	Mixed	Healthy subjects (Power, intermittent & endurance training)	rs762551 (CYP1A2)
**S175 -** Spineli H et al, 2024	Brazilian	Hispanic/Latin American	75	Male	Caffeine supplementation	I/D (ACE)
**S176 -** Tobina T et al, 2010	Japanese	East Asian	37+335	Male	>5000m runners	I/D (ACE)
**S177 -** Tural E et al, 2014	Turkish	Greater Middle Eastern	60+110	Mixed	Endurance (not specified)	rs4253778 (PPARa), rs8192678 (PPARGC1A)
**S178 -** Valdivieso P et al, 2017	British	European	28+24	Male	Healthy subjects (endurance training)	I/D (ACE)
**S179 -** van Breda K et al, 2015	Caucasian	European	51+56	Male	Ironman	rs4680 (COMT)
**S180 -** van Rossum EF et al, 2004	Caucasian	European	337	Mixed	Young Adults (Strength)	ER22/23EK (NR3C1)
**S181 -** Varillas-Delgado D et al, 2022	Not specified	Not reported/mixed	292+160	Male	Professional cycling/long-distance running and professional	rs3892097 (CYP2D6); present/null (GSTM1);

Study	Population	Ancestry	Sample size (Case + Control)	Gender	Sport discipline / phenotype	Polymorphisms / genetic approach
					football	rs1695 (GSTP1); present/null (GSTT1); rs1799945, rs1800562 (HFE); rs17602729 (AMPD1); rs8192678 (PPARGC1A); I/D rs4340 (ACE); rs2070744, rs1799983 (NOS3); rs1800544 (ADRA2A); rs1042713, rs1042714 (ADRB2); rs5810761 (BDKRB2); rs1815739 (ACTN3); rs8111989 (CKM); rs28497577, rs2700352 (MLCK); four TGS profiles
**S182 -** Varillas-Delgado D et al, 2023	Spanish	European	45+55	Mixed	Sports injuries	rs17602729 (AMPD1)
**S183 -** Voisin S et al, 2014	Polish & Russian	European	592+603	Mixed	Various sports disciplines	rs895436, rs1867782, rs11689011, rs4035887 (EPAS1)
**S184 -** Walsh S et al, 2009	Not specified	Not reported/mixed	754	Mixed	Healthy subjects (strength and endurance training)	rs1800169 (CNTF)
**S185 -** Wang G et al, 2013	Caucasian (European, Commonwealth, US & Russian cohorts)	East Asian; European	200+1248 / 193+1694	Mixed	Elite swimming, SMD and LD	I/D (ACE); rs1815739 (ACTN3)

Study	Population	Ancestry	Sample size (Case + Control)	Gender	Sport discipline / phenotype	Polymorphisms / genetic approach
**S186 -** Wang G et al, 2025	Jamaican, African-American, Japanese; European replication	African; East Asian; European	3 GWAS + 2 replication cohorts	Mixed	Sprint/power	rs10196189 (GALNT13); multi-ethnic GWAS
**S187 -** Wang Y et al, 2024	Chinese	East Asian	169+418 (training set)	Mixed	Sprint/power athlete status	2075 GWAS Catalog variants tested; 118-SNP and 30-SNP TGS models (no Bonferroni-significant individual marker)
**S188 -** Williams CJ et al, 2021	European	European	507	Mixed	GWAS	GWAS of VO2peak training response
**S189 -** Windelinckx A et al, 2007	Belgian	European	492	Mixed	Healthy subjects (Strength)	rs2228570, rs2544410, rs731236 (VDR)
**S190 -** Windelinckx A et al, 2011	Caucasian	European	145	Male	Healthy subjects (Strength)	rs2854464 (ACVR1B)
**S191 -** Wojciechowicz B et al, 2021	Polish	European	204+161	Mixed	Various sports disciplines	rs429358, rs7412 (APOE); rs20455 (KIF6)
**S192 -** Wolfarth B et al, 2007	Caucasian	European	313+297	Male	Various endurance disciplines	rs1042713 (ADRB2)
**S193 -** Xia X et al, 2014	Han Chinese	East Asian	102+206	Mixed	Long-distance and marathon runners	rs5418 (SLC2A4)
**S194 -** Yang R et al, 2017	Han Chinese	East Asian	103+50	Mixed	Various sports disciplines	rs1815739 (ACTN3)
**S195 -** Yang R et al, 2021	Chinese Han elite athletes	East Asian	60+43	Mixed	Various power and endurance disciplines	rs1815739 (ACTN3); rs4994 (ADRB3); rs8192678 (PPARGC1A)
**S196 -** Yang X et al, 2023	Chinese	East Asian	228	Mixed	VO2max response to 12-wk HIIT	rs17116985 (GABRB3)
**S197 -** Yvert T et al, 2012	Spanish & Han Chinese	East Asian; European	323+701	Mixed	Runners, road cyclists	rs6552828 (ACSL1)
**S198 -** Yvert T et al, 2016	Japanese	East Asian	60+649	Mixed	Runners	rs1815739 (ACTN3);

Study	Population	Ancestry	Sample size (Case + Control)	Gender	Sport discipline / phenotype	Polymorphisms / genetic approach
						rs5443 (GNB3)
**S199 -** Yvert T et al, 2020	Japanese	East Asian	106 women	Female	Vastus lateralis muscle-fiber composition	rs6949152 (NRF1); rs8192678 (PPARGC1A)
**S200 -** Yvert TP et al, 2018	Japanese, Russian & Polish	East Asian; European	282+2024	Mixed	Sprinters, jumpers and throwers	rs11091046 (AGTR2)
**S201 -** Zarębska A et al, 2013	Polish	European	100(P)+123(E) +354	Mixed	Triathletes, marathon, rowing, swimming of various distances, sprinters, powerlifting, weightlifting...	rs699 (AGT)
**S202 -** Zarebska A et al, 2014	Polish & Russian	European	1144+1540	Mixed	Various sports disciplines	rs1801131 (MTHFR)
**S203 -** Zmijewski P et al, 2018	Polish	European	60+157 / 50+379	Female	Elite swimming status	rs1799983, rs2070744 (NOS3)
**S204 -** Zmijewski P et al, 2021	Polish	European	196+379	Mixed	Swimmers of various distances	rs9939609 (FTO)
