# Supplementary material for: From Single Markers to Polygenic Panels: A Systematic Review of Genetics in Athletic Performance
Source: Sports (Basel). 2026 Oct 16;14(10):461. doi: 10.3390/sports14100461 (PMC13709855; doi:10.3390/sports14100461)
Supplement: Supplementary file 1 [file sports-14-00461-s001.zip › Supplementary Material S4. INPLASY Protocol 8850 (1).pdf]

International Platform of Registered Systematic Review and Meta-analysis Protocols

INPLASY

From single markers to polygenic panels: a global systematic review of genetics in athletic performance

INPLASY202630012

doi: 10.37766/inplasy2026.3.0012

Received: 4 March 2026

Published: 4 March 2026

Fernández-Lorenzo, S; Marín-Pagán, C.; Alcaraz, P.E.; Escobar, J; Martínez-Noguera, F.J.

Corresponding author:
Samuel Fernández Lorenzo

samuel.fernandez@sabartech.com

Author Affiliation:
Sabartech S.L - CIARD (UCAM).

ADMINISTRATIVE INFORMATION

Support - This research is being made possible within an Industrial Doctorate project between the High Performance Sports Research Center at the Catholic University of San Antonio in Murcia and Sabartech S.L. The funders have no role in the design of the studies, data collection and analysis, decision to publish or preparation of manuscripts.

Review Stage at time of this submission - The review has not yet started.

Conflicts of interest - Samuel Fernández Lorenzo is working on his Industrial Doctorate project at Sabartech S.L. in collaboration with UCAM and the High Performance Sports Research Centre. The rest of the researchers declare that they have no additional conflicts of interest with regard to this work.

INPLASY registration number: INPLASY202630012

Amendments - This protocol was registered with the International Platform of Registered Systematic Review and Meta-Analysis Protocols (INPLASY) on 4 March 2026 and was last updated on 4 March 2026.

INTRODUCTION

Review question / Objective Systematically identify and synthesise human studies that evaluate associations between DNA variants (candidate polymorphisms and genomic variants) and phenotypes related to athletic performance, including athlete status, objective measures of performance (endurance/power/strength), training response traits, or injury-related phenotypes in athletic populations.

Rationale Sports genomics has developed over the last few decades, providing information on the associations between genetic variants and athletic performance. However, results are often inconsistent across different sports, populations, and phenotypic definitions, and their replication is limited. A comprehensive, up-to-date synthesis is needed to (i) map the breadth of variants investigated, (ii) summarise the consistency of evidence across performance-related phenotypes and sporting disciplines, and (iii) highlight knowledge gaps and priorities for future research and polygenic approaches.

Condition being studied Athletic performance and performance-related phenotypes in humans (e.g., elite athlete status, endurance/power/strength performance outcomes, physiological indicators of performance such as VO_2max, responsiveness to training, and susceptibility to injury in cohorts of athletes). This review focuses on genetic variants that could contribute to inter-individual differences in these athletic performance phenotypes.

Fernández-Lorenzo et al. INPLASY protocol 202630012. doi:10.37766/inplasy2026.3.0012 Downloaded from https://inplasy.com/inplasy-2026-3-0012/

METHODS

Search strategy Electronic searches will be conducted in PubMed/MEDLINE and Web of Science from 1 January 1998 to 1 March 2026, limited to human studies in English and excluding preprints. Search terms will combine concepts related to sport and performance (e.g., sport, athlete, athletic performance, endurance, strength, power, VO2max) with concepts related to genetics (gene, variant, polymorphism). Example search strings will include combinations of PubMed titles/abstracts and a Web of Science title search: TI=((‘Sport’ OR ‘Endurance’ OR ‘Strength’ OR ‘Power’ OR ‘Athlete’ OR ‘Sports performance’) AND (‘Gene’ OR “Variant” OR ‘Polymorphism’)). A selection will be made from the reference list of the included studies to identify additional eligible records.

Participant or population Individuals of any gender, age, and ancestry, including athletes (elite, sub-elite, and recreational) and non-athlete controls, depending on the study design. Studies should include at least 20 participants per study and report genotype/allele and at least one phenotype related to athletic performance (or athlete status).

Intervention Not applicable.

Comparator Depending on the study design included: non-athletes/controls versus athletes (studies on the status of athletes); genotype/allele groups (e.g., AA versus AG versus GG) for performance or injury phenotypes; or comparisons between genotypes of pre- and post-changes in training response studies.

Study designs to be included Case-control studies (athletes versus controls); observational association studies linking genotype to performance tests/traits; training response studies (pre- and post-adaptations according to genotype); injury association studies in athlete cohorts; and genome-wide association studies (GWAS).

Eligibility criteria Inclusion: (i) original research in English on human subjects; (ii) published between 1998 and 1 March 2026; (iii) sample size ≥20; (iv) reports of at least one genetic variant and one phenotype related to athletic performance (athlete status, performance outcome/trait, response to training, or injury phenotype) with sufficient methodological detail and genotypic/allelic data.
Exclusion: animal studies; reviews/systematic reviews/meta-analyses; conference abstracts/letters/commentaries/interviews; studies lacking adequate methodological details, complete genotypic data, or relevant performance outcomes.

Information sources Electronic searches will be conducted in PubMed/MEDLINE and Web of Science. Additional records will be identified by reviewing the reference lists of included studies (back-citation search). The planned date for the final search is 1 March 2026 for each source.

Main outcome(s) Evidence of association between genetic variants and phenotypes related to athletic performance. The results will be classified by gene and variant, in addition to identifying the main phenotype in which associations have been found (power, endurance, injuries, etc.).

Data management Records will be exported from databases and managed in a structured spreadsheet/reference manager. Duplicates will be removed prior to selection. Selection decisions, reasons for exclusion from full text, and extracted data fields (study characteristics, sport/discipline, population, variant identifiers, outcomes, and key findings) will be recorded in standardised extraction sheets. Selection will be documented using a PRISMA 2020 flow diagram.

Quality assessment / Risk of bias analysis The methodological quality and quality of the reports will be assessed using criteria based on STROBE and STREGA. Two reviewers will independently assess the studies using a standardised checklist, and the individual scores of each reviewer will be averaged. A pre-established threshold (score ≥17) will be used as an inclusion filter.

Strategy of data synthesis The results will be classified according to genes and variants, indicating the affected genotypes and the direction of the effect. In addition, the disciplines or modalities affected in each case will be indicated. The results will be discussed in greater depth in the discussion section.

Subgroup analysis The analyses will be performed on the basis of each individual study. For each study, each variant analysed will be treated separately, indicating in each case the relevant information for each one: (I) name of the first author, (II) PMID (or, failing that, link to the digital object identifier (DOI)), (III) genes and polymorphisms investigated in the publications, (IV) ancestry of the study population, (V) sex, (VI) type of discipline (power, endurance, injuries, etc.), (VII) sport practised by the participants (athletics,

Fernández-Lorenzo et al. INPLASY protocol 202630012. doi:10.37766/inplasy2026.3.0012 Downloaded from https://inplasy.com/inplasy-2026-3-0012/

swimming, football, etc.), (VIII) number of participants, (IX) genotypic or allelic frequencies of the participants, and (X) brief description of the results obtained.

Sensitivity analysis If quantitative synthesis is undertaken, sensitivity analyses will include: excluding studies below the quality threshold or with key reporting limitations; restricting to larger samples; restricting to studies with comparable phenotype definitions; and restricting to a single ancestry group where population stratification is likely to influence results. If only narrative synthesis is feasible, sensitivity will be addressed by explicitly weighting interpretation toward higher-quality and better-replicated findings.

Language restriction Yes. Searches will be restricted to English-language publications.

Country(ies) involved Spain.

Other relevant information The review will follow PRISMA 2020 reporting guidance. Reference list screening (backward citation searching) will be used to identify additional eligible studies beyond database results. All search strings, screening logs, and extraction templates will be retained for transparency and reproducibility.

Keywords Athletic performance; sports genetics; polymorphism; genetic variant; GWAS; elite athletes; systematic review.

Dissemination plans The results will be submitted for publication in a peer-reviewed scientific journal on sports science, genetics or a similar field and may be presented at relevant scientific conferences. Supplementary materials (complete search strategies, PRISMA flow and quality scores) will be provided to ensure transparency and reproducibility.

Contributions of each author

Author 1 - Samuel Fernández Lorenzo - Conceptualization, Methodology, Investigation, Writing - Original Draft, Visualization.
Email: samuel.fernandez@sabartech.com
Author 2 - Cristian Marín Pagán - Methodology, Data Curation, Investigation.
Email: cmarin@ucam.edu
Author 3 - Pedro Emilio Alcaraz Ramón - Data Curation, Investigation.
Email: palcaraz@ucam.edu
Author 4 - Javier Escobar Cubiella - Writing - Review & Editing, Supervision.
Email: javier.escobar@sabartech.com
Author 5 - Francisco Javier Martínez Noguera - Conceptualization, Formal analysis, Writing - Review & Editing.
Email: fjmartinez3@ucam.edu

Fernández-Lorenzo et al. INPLASY protocol 202630012. doi:10.37766/inplasy2026.3.0012 Downloaded from https://inplasy.com/inplasy-2026-3-0012/
