# Supplementary material for: From Single Markers to Polygenic Panels: A Systematic Review of Genetics in Athletic Performance
Source: Sports (Basel). 2026 Oct 16;14(10):461. doi: 10.3390/sports14100461 (PMC13709855; doi:10.3390/sports14100461)
Supplement: Supplementary file 1 [file sports-14-00461-s001.zip › Supplementary Material S5. PRISMA_2020_Checklist.pdf]

PRISMA 2020 Checklist

Supplementary Material S5. Completed PRISMA 2020 Checklist

Manuscript: From single markers to polygenic panels: a systematic review of genetics in athletic performance

Page numbers refer to the submitted manuscript. Items specific to meta-analysis, formal statistical heterogeneity testing, sensitivity analysis, reporting-bias assessment, or certainty grading are marked not applicable because this review used a narrative synthesis.

Section and Topic	Item #	Checklist item	Location where item is reported
**TITLE**			
Title	1	Identify the report as a systematic review.	p. 1, title.
**ABSTRACT**			
Abstract	2	See the PRISMA 2020 for Abstracts checklist.	p. 1, Abstract.
**INTRODUCTION**			
Rationale	3	Describe the rationale for the review in the context of existing knowledge.	pp. 1-2, Section 1 (Introduction).
Objectives	4	Provide an explicit statement of the objective(s) or question(s) the review addresses.	p. 2, final paragraph of Section 1.
**METHODS**			
Eligibility criteria	5	Specify the inclusion and exclusion criteria for the review and how studies were grouped for the syntheses.	p. 3, Section 2.3 (Inclusion and Exclusion Criteria).
Information sources	6	Specify all databases, registers, websites, organisations, reference lists and other sources searched or consulted to identify studies. Specify the date when each source was last searched or consulted.	p. 2, Section 2.1 (Search Strategy); PubMed, Web of Science, and reference-list searching; final search date 1 March 2026.
Search strategy	7	Present the full search strategies for all databases, registers and websites, including any filters and limits used.	p. 2, Section 2.1; full database strategies, Boolean operators, filters, and search outputs in Supplementary Material 2.
Selection process	8	Specify the methods used to decide whether a study met the inclusion criteria of the review, including how many reviewers screened each record and each report retrieved, whether they worked independently, and if applicable, details of automation tools used in the process.	pp. 2-4, Sections 2.2 and 3.1; Figure 1; screening records in Supplementary Material 2.
Data collection process	9	Specify the methods used to collect data from reports, including how many reviewers collected data from each report, whether they worked independently, any processes for obtaining or confirming data from study investigators, and if applicable, details of automation tools used in the process.	p. 3, Section 2.4 (Data Extraction); standardized extracted fields and study records in Supplementary Materials 1 and 3.
Data items	10a	List and define all outcomes for which data were sought. Specify whether all results that were compatible with each outcome domain in each study were sought (e.g. for all measures, time points, analyses), and if not, the methods used to decide which results to collect.	pp. 2-3, Sections 2.3-2.4; athlete status, performance, training response, injury, and GWAS outcomes.
	10b	List and define all other variables for which data were sought (e.g. participant and intervention characteristics, funding sources). Describe any assumptions made about any missing or unclear information.	p. 3, Section 2.4; study, population, sex, discipline, sport, sample size, genotype/allele, and result variables; Supplementary Material 3.
Study risk of bias assessment	11	Specify the methods used to assess risk of bias in the included studies, including details of the tool(s) used, how many reviewers assessed each study and whether they worked independently, and if applicable, details of automation tools used in the process.	p. 3, Section 2.5 (Reporting Completeness Assessment); STROBE/STREGA, two independent reviewers, threshold >=17; study assessments in Supplementary Material 2.
Effect measures	12	Specify for each outcome the effect measure(s) (e.g. risk ratio, mean difference) used in the synthesis or presentation of results.	Not applicable: no meta-analysis or pooled effect measure was used; associations were synthesized narratively.
Synthesis methods	13a	Describe the processes used to decide which studies were eligible for each synthesis (e.g. tabulating the study intervention characteristics and comparing against the planned groups for each synthesis (item #5)).	pp. 3 and 5-16, Sections 2.3-2.4, 3.2, and 4; studies grouped by gene/variant and performance domain; Table 1 and Supplementary Materials 1 and 3.
	13b	Describe any methods required to prepare the data for presentation or synthesis, such as handling of missing summary statistics, or data conversions.	Not applicable: no conversion of summary statistics or quantitative data preparation was required.
	13c	Describe any methods used to tabulate or visually display results of individual studies and syntheses.	p. 3, Section 2.4; pp. 5-9, Table 1; Supplementary Materials 1 and 3.
	13d	Describe any methods used to synthesize results and provide a rationale for the choice(s). If meta-analysis was performed, describe the model(s), method(s) to identify the presence and extent of statistical heterogeneity, and software package(s) used.	pp. 5 and 10-16, Sections 3.2 and 4.1-4.10; narrative synthesis by biological pathway, gene/variant, and performance domain; no meta-analysis.
	13e	Describe any methods used to explore possible causes of heterogeneity among study results (e.g. subgroup analysis, meta-regression).	pp. 10-16, Discussion; narrative comparisons across power, endurance, intermittent/combat, injury, and population contexts; no formal meta-regression.
	13f	Describe any sensitivity analyses conducted to assess robustness of the synthesized results.	Not applicable: no formal sensitivity analysis was conducted because no quantitative synthesis was performed.
Reporting bias assessment	14	Describe any methods used to assess risk of bias due to missing results in a synthesis (arising from reporting biases).	Not applicable: reporting-bias assessment was not performed for the narrative synthesis.

PRISMA

PRISMA 2020 Checklist

Section and Topic	Item #	Checklist item	Location where item is reported
Certainty assessment	15	Describe any methods used to assess certainty (or confidence) in the body of evidence for an outcome.	A prespecified three-tier, design-based evidence-strength framework was used to classify gene–variant–phenotype associations and polygenic models according to replication, study design, genome-wide significance, multiple-testing correction, sample size, ancestry consistency and phenotype comparability (Section 2.6).
**RESULTS**			
Study selection	16a	Describe the results of the search and selection process, from the number of records identified in the search to the number of studies included in the review, ideally using a flow diagram.	pp. 3-4, Section 3.1 (Search Results) and Figure 1 (PRISMA 2020 flow diagram).
	16b	Cite studies that might appear to meet the inclusion criteria, but which were excluded, and explain why they were excluded.	p. 4, Section 3.1 (summary reasons); detailed screening/exclusion records in Supplementary Material 2.
Study characteristics	17	Cite each included study and present its characteristics.	pp. 5-9, Table 1; Supplementary Material 3 (included-study characteristics) and Supplementary Material 1.
Risk of bias in studies	18	Present assessments of risk of bias for each included study.	p. 3, Section 2.5; study-level STROBE/STREGA assessments in Supplementary Material 2.
Results of individual studies	19	For all outcomes, present, for each study: (a) summary statistics for each group (where appropriate) and (b) an effect estimate and its precision (e.g. confidence/credible interval), ideally using structured tables or plots.	pp. 5-16, Sections 3.2 and 4; Table 1; individual study information and findings in Supplementary Materials 1 and 3.
Results of syntheses	20a	For each synthesis, briefly summarise the characteristics and risk of bias among contributing studies.	pp. 5-16, Sections 3.2 and 4; Table 1 and Supplementary Materials 1-3.
	20b	Present results of all statistical syntheses conducted. If meta-analysis was done, present for each the summary estimate and its precision (e.g. confidence/credible interval) and measures of statistical heterogeneity. If comparing groups, describe the direction of the effect.	Not applicable to statistical synthesis: no meta-analysis was conducted. Narrative synthesis results are reported on pp. 5-16.
	20c	Present results of all investigations of possible causes of heterogeneity among study results.	No formal heterogeneity analysis was conducted; narrative comparisons by phenotype, discipline, and population are reported on pp. 10-16.
	20d	Present results of all sensitivity analyses conducted to assess the robustness of the synthesized results.	Not applicable: no sensitivity analyses were conducted.
Reporting biases	21	Present assessments of risk of bias due to missing results (arising from reporting biases) for each synthesis assessed.	Not applicable: risk of bias due to missing results was not formally assessed.
Certainty of evidence	22	Present assessments of certainty (or confidence) in the body of evidence for each outcome assessed.	Evidence-strength classifications are presented in Table 1 and applied throughout Section 4. These tiers represent methodological strength and degree of replication rather than a formal GRADE certainty rating.
**DISCUSSION**			
Discussion	23a	Provide a general interpretation of the results in the context of other evidence.	pp. 10-16, Section 4 (Discussion), including comparison with previous reviews and meta-analyses.
	23b	Discuss any limitations of the evidence included in the review.	p. 16, Section 4.11 (Strengths and Limitations).
	23c	Discuss any limitations of the review processes used.	p. 16, Section 4.11 (Strengths and Limitations).
	23d	Discuss implications of the results for practice, policy, and future research.	pp. 16-17, Section 5 (Conclusions), including implications for future polygenic and multi-omics research and cautious practice.
**OTHER INFORMATION**			
Registration and protocol	24a	Provide registration information for the review, including register name and registration number, or state that the review was not registered.	p. 3, Section 2.7; p. 17, Supplementary Materials statement. INPLASY registration: INPLASY202630012.
	24b	Indicate where the review protocol can be accessed, or state that a protocol was not prepared.	p. 17; protocol supplied as Supplementary Material 4.
	24c	Describe and explain any amendments to information provided at registration or in the protocol.	During peer-review revision, three supplementary PubMed search strategies were added to improve sensitivity for genome-wide, sequencing-based, polygenic, cardiorespiratory-fitness and elite-athlete association studies. The supplementary searches covered the same publication period and core review question as the original strategy. The amendment is described in Sections 2.1 and 2.8 and documented in Supplementary Material 2.
Support	25	Describe sources of financial or non-financial support for the review, and the role of the funders or sponsors in the review.	p. 17, Funding and Acknowledgments.
Competing interests	26	Declare any competing interests of review authors.	p. 17, Conflicts of Interest.
Availability of data, code and other materials	27	Report which of the following are publicly available and where they can be found: template data collection forms; data extracted from included studies; data used for all analyses; analytic code; any other materials used in the review.	p. 17, Data Availability Statement and Supplementary Materials statement; supporting data/materials in Supplementary Materials 1-4.

From: Page MJ, McKenzie JE, Bossuyt PM, Boutron I, Hoffmann TC, Mulrow CD, et al. The PRISMA 2020 statement: an updated guideline for reporting systematic reviews. BMJ 2021;372:n71. doi: 10.1136/bmj.n71. This work is licensed

PRISMA 2020 Checklist

under CC BY 4.0. To view a copy of this license, visit https://creativecommons.org/licenses/by/4.0/
