# Supplementary material for: From Single Markers to Polygenic Panels: A Systematic Review of Genetics in Athletic Performance
Source: Sports (Basel). 2026 Oct 16;14(10):461. doi: 10.3390/sports14100461 (PMC13709855; doi:10.3390/sports14100461)
Supplement: Supplementary file 1 [file sports-14-00461-s001.zip › New - Supplementary Material S2.zip!/NEW - Search strategy.pdf]

Search strategy

1. Entries in PubMed and Web of Science

Date searched (last searched): 05/03/2026

1.1. PubMed 1

Entry: (("Sport"[Title/Abstract] OR "Athlete"[Title/Abstract] OR "athletic performance"[Title/Abstract]) AND ("Polymorphism"[Title/Abstract] OR "Variant"[Title/Abstract] OR "Gene"[Title/Abstract]) AND (("all"[Filter] NOT "preprint"[Publication Type]) AND ("clinical study"[Publication Type] OR "clinical trial"[Publication Type] OR "meta analysis"[Publication Type] OR "observational study"[Publication Type] OR "randomized controlled trial"[Publication Type] OR "review"[Publication Type] OR "systematic review"[Filter]) AND "humans"[MeSH Terms] AND "english"[Language])) AND ((excludepreprints[Filter]) AND (clinicalstudy[Filter] OR clinicaltrial[Filter] OR meta-analysis[Filter] OR observationalstudy[Filter] OR randomizedcontrolledtrial[Filter] OR review[Filter] OR systematicreview[Filter]) AND (humans[Filter]) AND (english[Filter]))

Publication date: From 1998/1/1 to 2026/3/1

Articles: 225

1.2. PubMed 2

Entry: (("Power"[Title/Abstract] OR "Strength"[Title/Abstract] OR "Endurance"[Title/Abstract] OR "VO2max"[Title/Abstract]) AND ("Polymorphism"[Title/Abstract] OR "Variant"[Title/Abstract] OR "Gene"[Title/Abstract]) AND ("SPORT"[Title/Abstract] OR "Athletic Performance"[Title/Abstract]) AND (("all"[Filter] NOT "preprint"[Publication Type]) AND ("clinical study"[Publication Type] OR "clinical trial"[Publication Type] OR "meta analysis"[Publication Type] OR "observational study"[Publication Type] OR "randomized controlled trial"[Publication Type] OR "review"[Publication Type] OR "systematic review"[Filter]) AND "humans"[MeSH Terms] AND "english"[Language]) AND (("all"[Filter] NOT "preprint"[Publication Type]) AND ("clinical study"[Publication Type] OR "clinical trial"[Publication Type] OR "meta analysis"[Publication Type] OR "observational study"[Publication Type] OR "randomized controlled trial"[Publication Type] OR "review"[Publication Type] OR "systematic review"[Filter]) AND "humans"[MeSH Terms] AND "english"[Language]) AND (("all"[Filter] NOT "preprint"[Publication Type]) AND ("clinical study"[Publication Type] OR "clinical trial"[Publication Type] OR "meta analysis"[Publication Type] OR "observational study"[Publication Type] OR "randomized controlled trial"[Publication Type] OR "review"[Publication Type] OR "systematic review"[Filter]) AND "humans"[MeSH Terms] AND "english"[Language])) AND ((excludepreprints[Filter]) AND (clinicalstudy[Filter] OR clinicaltrial[Filter] OR meta-analysis[Filter] OR observationalstudy[Filter] OR randomizedcontrolledtrial[Filter] OR review[Filter] OR systematicreview[Filter]) AND (humans[Filter]) AND (english[Filter]))

Publication date: From 1998/1/1 to 2026/3/1

Articles: 64

1.3. PubMed 3

Entry: ("genes"[Title/Abstract] AND "athletic performance"[Title/Abstract]) AND ((excludepreprints[Filter]) AND (humans[Filter]) AND (english[Filter]))

Publication date: From 1998/1/1 to 2026/3/1

Articles: 97

1.4. PubMed 4

Entry: (("athlete*"[Title/Abstract] OR "swimmer*"[Title/Abstract] OR "sprinter*"[Title/Abstract] OR "runner*"[Title/Abstract] OR "cyclist*"[Title/Abstract] OR "athletic performance"[Title/Abstract] OR "sports performance"[Title/Abstract] OR "athlete status"[Title/Abstract] OR "physical performance"[Title/Abstract]) AND ("GWAS"[Title/Abstract] OR "genome-wide association"[Title/Abstract] OR "genome-wide association"[Title/Abstract] OR "whole-genome sequencing"[Title/Abstract] OR "whole-genome sequencing"[Title/Abstract] OR "whole-exome sequencing"[Title/Abstract] OR "whole-exome sequencing"[Title/Abstract] OR "polygenic"[Title/Abstract] OR "polygenic score"[Title/Abstract] OR "polygenic risk score"[Title/Abstract] OR "genetic score"[Title/Abstract] OR "genotype score"[Title/Abstract] OR "total genotype score"[Title/Abstract] OR "genetic profile"[Title/Abstract]) AND 1998/01/01:2026/03/01[Date - Publication] AND "english"[Language]) AND ((excludepreprints[Filter]) AND (humans[Filter]) AND (1998/1/1:2026/3/1[pdat]) AND (english[Filter]))

Publication date: From 1998/1/1 to 2026/3/1

Articles: 148

1.5. PubMed 5

Entry: (("cardiorespiratory fitness"[Title/Abstract] OR "VO2max"[Title/Abstract] OR "VO2 max"[Title/Abstract] OR "VO2peak"[Title/Abstract] OR "VO2 peak"[Title/Abstract] OR "maximal oxygen uptake"[Title/Abstract] OR "peak oxygen uptake"[Title/Abstract] OR "aerobic capacity"[Title/Abstract]) AND ("GWAS"[Title/Abstract] OR "genome-wide association"[Title/Abstract] OR "genome-wide association"[Title/Abstract] OR "polygenic"[Title/Abstract] OR "polygenic score"[Title/Abstract] OR "polygenic risk score"[Title/Abstract] OR "genetic score"[Title/Abstract] OR "genotype score"[Title/Abstract]) AND 1998/01/01:2026/03/01[Date - Publication] AND "english"[Language]) AND ((excludepreprints[Filter]) AND (humans[Filter]) AND (1998/1/1:2026/3/1[pdat]) AND (english[Filter]))

Publication date: From 1998/1/1 to 2026/3/1

Articles: 39

1.6. PubMed 6

Entry: (("elite"[Title] AND ("athlete*"[Title] OR "swimmer*"[Title] OR "sprinter*"[Title] OR "runner*"[Title] OR "cyclist*"[Title] OR "rower*"[Title] OR "weightlift*"[Title] OR "sprint*"[Title] OR "power"[Title] OR "endurance"[Title]) AND ("associat*"[Title] OR "polymorphism*"[Title] OR "genotype*"[Title] OR "variant*"[Title] OR "snp"[Title] OR "GWAS"[Title] OR "genome-wide association"[Title]) AND ("performance"[Title] OR "predisposition"[Title] OR "athlete status"[Title/Abstract] OR "athletic status"[Title/Abstract] OR "swimmer status"[Title/Abstract] OR "elite status"[Title/Abstract]) AND 1998/01/01:2026/03/01[Date - Publication]) NOT "preprint"[Publication Type]) NOT "review"[Publication Type]

Publication date: From 1998/1/1 to 2026/3/1

Articles: 88

Total PubMed

661 articles

1.7. Web Of Science

Entry: TI=(("Sport" OR "Endurance" OR "Strength" OR "Power" OR "Athlete" OR "Athletic Performance") AND ("Gene" OR "Variant" OR "Polymorphism"))

Filters:

- 1998-01-01 to 2026-03-01
- Refined by:
    - Languages: English
    - Document Types: Article
    - Web of Science Categories: Sport Sciences, Genetics Heredity, Physiology, Biochemistry Molecular Biology

664 articles

2. Removal of duplicates among PubMed entries

2.1. Total duplicates

137 duplicates

2.2. Total non-duplicates

524 total non-duplicates

3. Unification of PubMed and WoS outputs

3.1. Duplicates between PubMed and WoS outputs

46 articles

3.2. Total non-duplicates

1142 articles in total

4. Manual addition of articles

4.1. Manually entered articles

6 articles

4.2. Complete list of unified articles

1148 articles in total

5. Filtered by title:

5.1. Elimination of meta-analyses and reviews

66 articles

5.2. Discards by title

688 articles

5.3. List of remaining articles

394 articles in total

6. Filtered by abstract:

6.1. Removal of remaining meta-analyses and reviews

56 articles

6.2. Reject due to abstract

28 articles

6.3. Articles in other languages

3 articles

6.4. Articles without access

16 articles

6.5. List of remaining items

291 articles

7. Article review:

7.1. Articles STREGA < 17

83 articles

7.2. Reject after reviewing articles

4 articles

7.3. Articles finally included in the review

204 articles in total
